# Modification of the Zeolite Heulandite with N-(3-Triethoxysilylpropyl)guanidines Offers an Effective Approach to Enhancing Its Adsorption Capacity for Heavy Metal Ions

**DOI:** 10.3390/ijms26167903

**Published:** 2025-08-15

**Authors:** Sergey N. Adamovich, Arailym M. Nalibayeva, Yerlan N. Abdikalykov, Mirgul Zh. Turmukhanova, Elena G. Filatova, Alexandr D. Chugunov, Igor A. Ushakov, Elizaveta N. Oborina, Igor B. Rozentsveig, Francis Verpoort

**Affiliations:** 1 E. Favorsky Irkutsk Institute of Chemistry, SB RAS, 1 Favorsky Str., 664033 Irkutsk, Russia; 2 D.V. Sokolsky Institute of Fuel, Catalysis and Electrochemistry, 142 D. Kunaeva Str., Almaty 050010, Kazakhstan; a.nalibayeva@ifce.kz (A.M.N.);; 3 Faculty of Chemistry and Chemical Technology, Al-Farabi Kazakh National University, 71 Al-Farabi Avenue, Almaty 050040, Kazakhstan; t_mirgul@mail.ru; 4 Department of Chemistry, Irkutsk National Research Technical University, 83 Lermontov Str., 664074 Irkutsk, Russia; 5 State Key Laboratory of Advanced Technology for Materials Synthesis and Processing, Wuhan University of Technology, 122 Luoshi Road, Wuhan 430070, China; francis@whut.edu.cn; 6 Joint Institute of Chemical Research (FFMiEN), Peoples Friendship University of Russia (RUDN University), 6 Miklukho-Maklaya Str., 117198 Moscow, Russia

**Keywords:** natural zeolite, silanes, modification, adsorption, Cu(II), Co(II), Ni(II) ions

## Abstract

Zeolites are widely used as adsorbents due to their porous structure and ion-exchange capabilities. However, their adsorption efficiency for heavy metal ions remains limited. To enhance their performance, the natural zeolite heulandite (**Z**) was functionalized with guanidine derivatives: N-[3-(triethoxysilyl)propyl]guanidine (**1**), -aminoguanidine (**2**), and -acetyl-guanidine (**3**). The resulting materials (**Z1**–**Z3**) were evaluated for their ability to adsorb Co^2+^, Cu^2+^, and Ni^2+^ from aqueous solutions. The composition and structure of silanes **1**–**3** were confirmed by FT-IR and ^1^H and ^13^C NMR spectroscopy methods. The modified zeolites were characterized using nitrogen adsorption/desorption (BET) and SEM-EDX to confirm their functionalization and assess the structural changes. A TGA-DSC was used to determine the thermal stability. The adsorption experiments were conducted in single and multi-ionic aqueous solutions at pH 5.0 to evaluate metal uptake. Functionalization significantly improved the adsorption efficiency, with **Z1**–**Z3** showing a three to six times greater adsorption capacity than the unmodified zeolite. The adsorption efficiency followed the trend Cu^2+^ > Co^2+^ > Ni^2+^, primarily due to chelate complex formation between the metal ions and guanidine groups. The SEM-EDX confirmed the co-localization of nitrogen atoms and metal ions. The functional materials (**Z1**–**Z3**) exhibited strong potential as adsorbents for noble, heavy, and toxic metal ions, and could find applications in industry, agriculture, ecology, medicine, chemistry, wastewater treatment, soil remediation, chemisorption, filtration, chromatography, etc.

## 1. Introduction

The pollution of water reservoirs with industrial, agricultural, and household waste containing heavy metal ions (HMIs) represents an urgent environmental challenge [1,2,3,4,5,6,7,8,9]. One of the effective methods for the purification of wastewater (WW) from (HMIs) is sorption purification. Various carbon, synthetic [10,11,12,13,14], and organic [15,16,17,18] materials, including biosorbents [19,20,21], are used as adsorbents. Among them, different types of mineral adsorbents are the most popular. At the moment, there are more than 100 modifications of such adsorbents, which differ in their structure, composition, properties, and applications [22,23,24,25,26,27,28,29,30,31].

However, cheap and available adsorbents, such as natural zeolites, are of particular importance in the purification of WW from HMIs [32,33,34]. At the same time, exchange sites of natural zeolites are located only on their external surface, which decreases sorption capacity of zeolites. In addition, the adsorbents employed for the purification of WW from HMIs should meet a number of requirements, e.g., hydrophobicity, thermal stability, regenerability, and mechanical stability. These properties can be achieved via the modification of natural zeolites.

Chemical modification can involve acid–base treatments [35,36,37,38,39,40]; intercalation; and functionalization through ion exchange with salts [41,42], rare earth metals [43], surfactants [44,45,46], and organic and organosilicon compounds capable of ion exchange or complex formation [47,48,49]. Hybrid adsorbents with stable technical characteristics and a high sorption capacity can be obtained by fixation of organic products bearing S- or O-functionality on the zeolite surface.

Zeolite surface modification using nitrogen-containing compounds enables the development of a branched surface, leading to enhanced sorption capacity and selectivity of adsorbent. Both polymers and low-molecular-weight nitrogen-containing compounds are capable of complex formation with HMIs. Therefore, they can be used to design adsorbents for the purification of WW. For instance, natural clinoptilolite zeolite (from the Sibay and Kholinsky deposits) modified with monoethanolamine was utilized to remove Cu(II) and Ni(II) from technogenic solutions [50]. Natural heulandite can be modified with hexamethylenediamine [51]. In addition, we recently successfully modified heulandite using a Si–organic derivative of thiosemicarbazide [52].

Guanidine, H_2_N-C(NH)-NH_2_, is known to be one of the simplest nitrogen-containing compounds. Guanidine and its derivatives have bactericidal and fungicidal activity. Recently, such compounds were proposed for the treatment of cancer and COVID-19 [53,54]. Due to its structural peculiarities and distribution of electron density, guanidine might be employed as a ligand in the formation of metal complexes [55]. It was reported that guanidine derivatives have a high affinity for hydrogen ions, as well as for ions of heavy and toxic metals (Co(II), Zn (II), and As(V)) [56].

In terms of their practical applications, the organosilicon derivatives of guanidine are of great interest. In this regard, we synthesized derivatives of guanidine, aminoguanidine, and acetylguanidine, namely N-[3-(triethoxysilyl)propyl]guanidine (**1**), N-[3-(triethoxysilyl)propyl]-aminoguanidine (**2**), and N-[3-(triethoxysilyl)propyl]acetylguanidine (**3**).

Heulandite (**Z**), a natural zeolite, was exploited as a carrier due to its accessibility and low price. Silanes **1**, **2**, and **3** were immobilized on zeolite **Z** to enhance its selectivity and adsorption capacity for extraction of Cu(II), Co(II), and Ni(II) from water solutions.

The experimental results showed that the zeolite **Z** (hereinafter **Z1**, **Z2**, and **Z3**) modified with silanes **1**, **2**, and **3** demonstrated a pronounced efficacy in the processes of Cu(II), Co(II), and Ni(II) extraction. The modified adsorbent can be used to remove impurities and heavy metal ions from water objects. To our knowledge, compounds of this type have not been employed previously for the modification of zeolites.

## 2. Results and Discussion

### 2.1. Guanidine-Containing Silanes

**Scheme 1:**
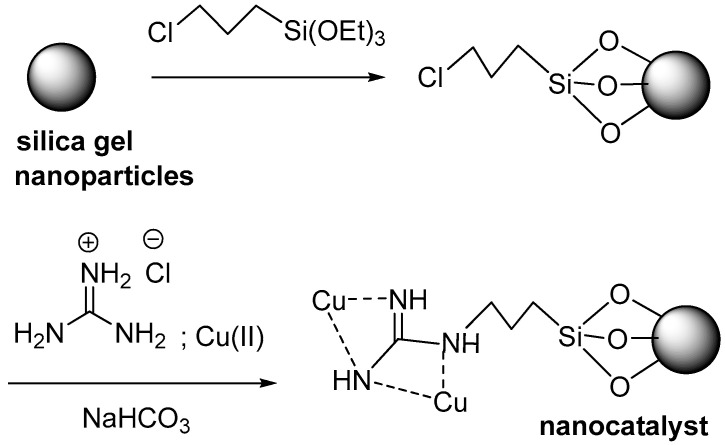
Synthesis of guanidine–Cu(II) catalyst nanoparticles.

Currently, organosilicon derivatives of guanidine are still rare compounds. Meanwhile, they may be of significant interest, since such silanes can be used as platforms for the design of special monomer and polymer materials possessing good film-forming and complexing properties, as well as high biological activity. In particular, this was reported for the synthesis of guanidine-containing silane, which turned out to be a universal, highly efficient, and reusable heterogeneous catalyst (Scheme 1) [57].

**Scheme 2:**
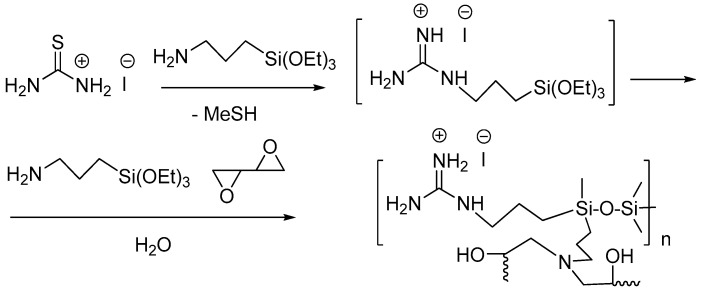
Organosilicon guanidine-containing epoxy material.

Guanidine–carbosilane dendrimers were recently prepared. It was found that their combination with chlorhexidine had a significant synergistic effect against trophozoites (malaria) [58]. The “Indirect” (via thiourea ylide and amino derivative) synthesis of guanidinopropyltriethoxysilane, its homopolymer, and epoxy compositions was described [59] (Scheme 2).

These guanidine-containing polysiloxanes and materials were shown to exhibit pronounced biocidal action with respect to *S. aureus*, *E. coli*, and *C. albicans* strains.

**Scheme 3:**
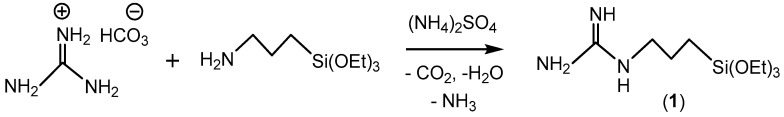
Synthesis of N-[3-(triethoxysilyl)propyl]guanidine (**1**); cat: (NH_4_)_2_SO_4_.

**Scheme 4:**
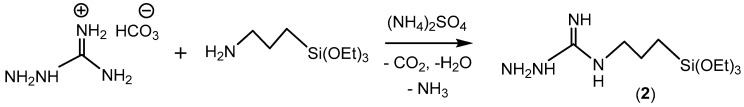
Synthesis of N-[3-(triethoxysilyl)propyl]amino guanidine (**2**); cat: (NH_4_)_2_SO_4_.

**Scheme 5:**
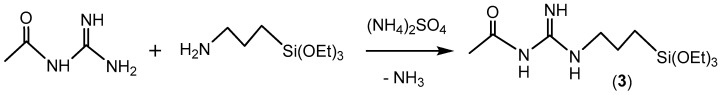
Synthesis of N-[3-(triethoxysilyl)propyl]acetyl guanidine (**3**); cat: (NH_4_)_2_SO_4_.

In the present work, N-[3-(triethoxysilyl)propyl]guanidines (**1**–**3**) were obtained directly from guanidines and 1-(3-aminopropyl)triethoxysilane (Scheme 3, Scheme 4 and Scheme 5). The synthesis was carried out as follows: a mixture of silane and the corresponding guanidine (1:1) in the presence of a catalytic amount of (NH_4_)_2_SO_4_ (in an argon atmosphere) was heated in a flask at 150 °C for 2 h until ammonia stopped being released. Silanes **1**–**3** are viscous liquids with b.p.~190–200 °C (0.3 mm Hg). The yields of silanes were 81–87%.

### 2.2. Immobilization of Guanidine–Silanes ***1***–***3*** on Zeolite

**Scheme 6:**
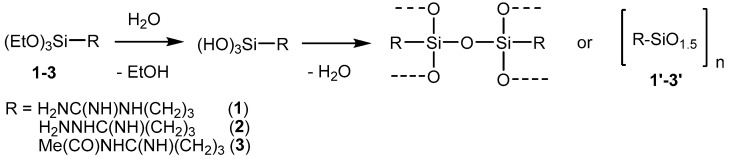
Formation of polyorganylsilsesquioxanes **1**––**3**– (homo-condensation reaction).

It should be noted that silanes **1**–**3** are unstable and, under the action of air moisture, undergo rapid hydrolysis and homo-condensation to produce the corresponding polyorganyl-silsesquioxanes **1**′–**3**′ (Scheme 6).

**Scheme 7:**
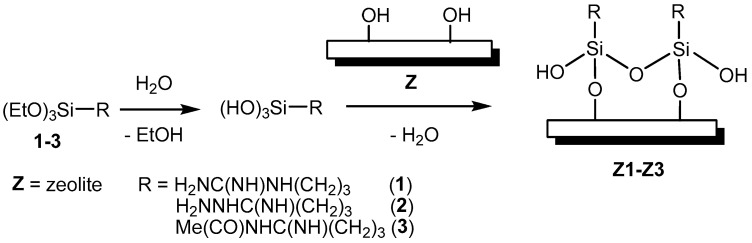
Formation of modified zeolites **Z1**, **Z2**, and **Z3** (hetero-condensation reaction).

To avoid such homo-condensation, silanes **1**–**3** were immobilized immediately after preparation on the hydroxylated surface of the zeolite **Z**. In this case, a hetero-condensation reaction occurred to produce self-assembled layers and the modified zeolites **Z1**, **Z2**, and **Z3** (Scheme 7).

The initial zeolite **Z** represents a polydisperse compound containing particles of irregular shape and different sizes. Consequently, the zeolite underwent preliminary fractionation. In the runs, a 0.5–1.0 mm fraction of the zeolite was employed. The synthesized silanes **1**–**3** were immobilized on the zeolite surface as described previously [52].

The degree of zeolite **Z** modification with the guanidine–silanes **1**–**3** was evaluated according to the increase in the modified zeolites’ **Z1**’**s**–**Z3**’**s** masses. From Table 1, it can be seen that the content of silanes **1**–**3** in the **Z** grew linearly with an increasing concentration of the silane solution. The best result was shown by **Z3**.

The grafting of silanes **1**–**3** onto the zeolite significantly changed its surface characteristics. For example, the treatment of zeolite **Z** with guanidine–silanes **1**–**3** reduced the specific pore volume, specific surface area, and micropore volume of the compound. The BET analysis data for the starting **Z** and modified **Z1**–**Z3** are given in Table 2.

### 2.3. TGA-DSC Analysis

**Figure 1:**
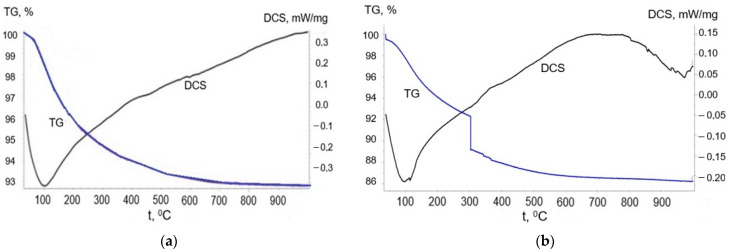
TG/DSC analysis of (**a**) **Z**; (**b**) **Z3**.

Figure 1 shows the results of TGA-DSC analysis. Upon the heating of original zeolite **Z**, the endothermic effect, which is caused by the removal of adsorbed water, appeared at 35–1000 °C. This was supported by the detection of a signal (*m*/*z* = 18). In this case, four stages of water removal were observed: at 35–58 °C, 58–362 °C, 362–444 °C, and 444–1000 °C. The heat curve also indicates endothermic effects due to the rearrangement of zeolite at 455, 561, 597, and 629 °C. After heating was completed, the residual mass of Z was 92.77%. The loss of mass was 7.23% (Figure 1a).

For the modified sample **Z3**, the endothermic effect was detected at 40–200 °C (Figure 1b). Due to the release of adsorbed water, the weight loss was 5.91%. This was evidenced by the signal *m*/*z* = 18 in the MS. As seen from the TG curve, the mass loss of sample **Z3**, from 92 to 89%, occurred at the same temperature—300 °C. This fact indicates that at 300 °C sample **Z3** was instantly decomposed. In this case, it was impossible to detect the temperature-dependent changes in mass.

After the performance of thermal analysis, the mass of **Z3** was found to be 86.19% of the initial. Thus, the weight loss was 13.81%. The weight loss difference between the modified **Z3** and the original **Z** (13.81 − 7.23% = 6.58%) evidenced the content of guanidine modifier **3** in **Z3**. The content of **3** in **Z3** was calculated to be 5.25%.

The results obtained are in good agreement with the literature data on the thermal analysis of modified natural zeolites (see Ref. [52] and the literature cited therein).

### 2.4. Adsorption Isotherms

As previously established, the original zeolite **Z** showed low adsorption activity (water, pH = 5). For instance, the values of adsorption capacity with respect to ions of Ni(II), Co(II), and Cu(II) were 0.10 mmol/g (5.9 mg/g), 0.095 mmol/g (5.6 mg/g), and 0.08 mmol/g (4.8 mg /g), correspondingly [60]. The adsorption activity of zeolite **Z** decreased as follows: Ni(II) > Co(II) > Cu(II). The adsorption capacity values correlated with the values of ionic radii, which were augmented as follows: Ni(II) < Co(II) < Cu(II). This indicates that in the case of microporous zeolites like **Z**, a smaller ionic radius corresponds with a better adsorption capacity [61]. However, for modified zeolites, these regularities may be different.

**Figure 2:**
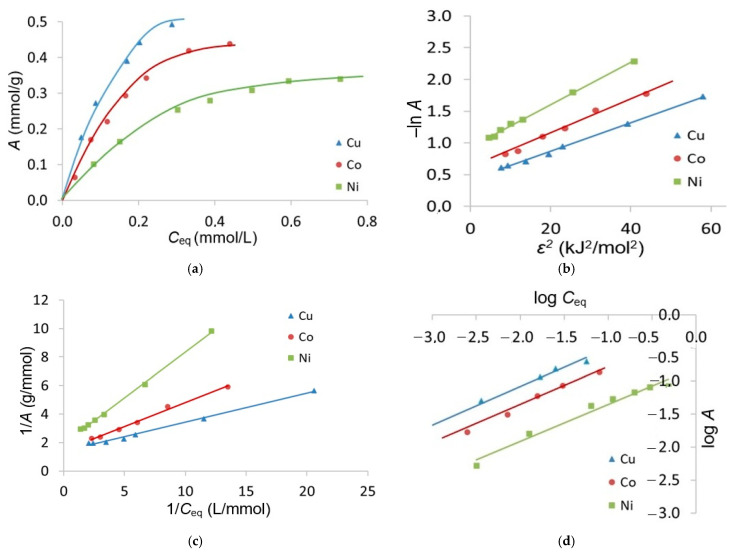
**Z3**: (**a**) adsorption isotherm, (**b**) Dubinin–Radushkevich adsorption isotherm, (**c**) Langmuir adsorption isotherm, (**d**) Freundlich adsorption isotherm (*C*_Cu_, *C*_Co_, *C*_Ni_: 5, 10, 20, 35, 50, 70, 90, 100 mg/L; sorbent 1 g/L; time 2 h; pH = 5–5.5; 298 K).

In the present paper, we investigated the adsorption characteristics of modified zeolites **Z1**, **Z2**, and **Z3**. Thus, the adsorption properties of **Z1**–**Z3** (water, pH = 5) relative to the ions of Cu(II), Co(II), and Ni(II) were evaluated by analyzing the adsorption isotherms. The best adsorption characteristics were demonstrated by **Z3** (Figure 2).

The values of adsorption capacity of **Z3** were 0.51 mmol/g (32.5 mg/g), 0.44 mmol/g (25.9 mg/g), and 0.34 mmol/g (20.0 mg/g) for Cu(II), Co (II), and Ni(II), correspondingly (Figure 2a). The capacity for **Z1** and **Z2** was 0.45, 0.39, and 0.30 mmol/g and 0.47, 0.41, and 0.32 mmol/g, respectively.

As follows from the literature survey, the adsorption capacities of zeolites **Z1**–**Z3** for Cu(II), Ni(II) and Co(II) exceed those for zeolites modified with both organic and organosilicon materials [62,63].

Thus, the modification of zeolite **Z** with silane **3** increased its adsorption activity with respect to the ions of Cu(II), Co(II), and Ni(II) by 6.4, 4.6, and 3.4 times, respectively, and the adsorption activity was augmented as follows: Ni(II) < Co(II) < Cu(II).

In an adsorption system, the equilibrium is known to depend on the interaction between the adsorbent and adsorbate [64]. The models of adsorption proposed by Freundlich, Langmuir, Dubinin–Radushkevich, etc., analyze such interactions differently.

Hence, it is rational to assess the suitability of the aforementioned models in order to interpret the obtained results (Figure 2b–d). The isotherms were plotted with the help of Dubinin–Radushkevich adsorption equation in the linear form of dependence ln *A* = *f* (ε^2^) (Figure 2b).

The constants *k* and *A*_m_ were established from the straight-line slope and the segment cut off along the ordinate axis, respectively. The Dubinin–Radushkevich model indicates the nature of the adsorbent adsorption on the adsorbate and can be employed to calculate the absorption free energy:

*E* = (−2*k*)^−0.5^ (1)

The *E* value is used for a mechanistic rationalization of the absorption. If *E* is changed from 8 to 16 kJ/mol, this indicates ion exchange or chemical processes [64]. When the *E* value is less than 8 kJ/mol, the adsorption is of physical nature. The obtained *E* values are shown in Table 3. The values of adsorption free energy are 14.9, 13.1, and 12.3 kJ/mol for Cu(II), Co(II), and Ni(II), and the *E* values are 8–16 kJ/mol. These evidence that the ions of heavy metals were adsorbed by **Z3** through ion exchange on the adsorbent surface or via the reactions with **Z3** functionalities. Here, the higher values of the adsorption energy *E* led to a larger maximum adsorption (Table 3).

The limiting adsorption *A*_∞_ was determined from the linear dependences (Figure 2c) on the basis of y-intercept, while the adsorption equilibrium constant *K* was calculated from the slope. The data obtained are shown in Table 3. The maximum monolayer capacity (*A*_∞_) of the Langmuir isotherm model was found to be 0.722, 0.691, and 0.520 mmol/g for Cu(II), Co(II), and Ni(II), correspondingly. The *R^2^* values of 0.994–0.999 indicate that the data on adsorption correlate well with this model.

The Δ*G*^0^ values are given in Table 3. Negative Δ*G*^0^ values point to the spontaneous elimination of heavy metal ions. It is known that Δ*G*^0^ values up to −20 kJ/mol indicate physical adsorption, which takes place owing to the electrostatic interaction of the adsorption cites with metal ions, while Δ*G*^0^ values from −40 kJ/mol denote chemical adsorption. The values of Δ*G*^0^—−21.82, −20.70, and −20.00 kJ/mol for Cu(II), Co(II), and Ni(II)— range from −20 to −40 kJ/mol, indicating probable ion exchange and chemical adsorption through charge separation or transfer from the **Z3** surface to cations of Cu(II), Co(II), and Ni(II) to form a coordination bond [65].

The *K*_F_ and *n* constants of the Freundlich equation allow for a comparison of the adsorption capacity of the modified samples. When the concentration of the ions in a solution is 1 mol/L, the adsorption value of these ions is equal to the constant *K*_F_. As can be seen from Table 3, the *K*_F_ value for the ions tends to 1. The parameter *n* indicates the intensity of the adsorbent–adsorbate interaction. The *n* coefficient values of 1.954, 1.724, and 1.481 point to a quite good intensity of the adsorption for the whole set of metal ion concentrations. Table 3 shows that the adsorption of Cu(II), Co(II), and Ni(II) ions is adequately described by the Freundlich isotherm. High *R*^2^ values were obtained for all the samples in question. Therefore, it can be concluded that the adsorption of heavy metal ions took place on a heterogeneous surface via a complex mechanism.

Thus, all the aforementioned models confirm that the adsorption activity was augmented as follows: Ni(II) < Co(II) < Cu(II).

### 2.5. FT-IR Spectroscopy

The FT-IR spectra of modified zeolites **Z1**–**Z3** were also recorded (Figures S1–S3). In the spectra of **Z1**–**Z3**, characteristic bands of silicate compounds were observed [60]. The bands of stretching vibrations ν_s_ 780–799 cm^−1^ and ν_as_ 1079–1085 cm^−1^ are associated with the symmetric and asymmetric stretching vibrations of SiO_4_ and AlO_4_ bonds in the primary structural units of zeolites. The bands of stretching vibrations of guanidine fragments in the samples of the modified zeolites **Z1** and **Z2**, ν (C–N) and ν (C=N), were observed at 1370–1400 cm^−1^ and 1627–1633 cm^−1^, respectively. Stretching ν (NH) and bending δ (NH, NH_2_) vibrations appeared at 3300–3400 and 1500–1550 cm^−1^, respectively. It is noteworthy that in the spectrum of zeolite **Z3**, modified with acetylguanidine–silane, a band at 1651 cm^−1^ (C=O) was detected. Thus, the FT-IR spectroscopy confirms the deposition of guanidine–silanes **1**–**3** on the surface of the zeolite **Z**.

The coordination C=N–metal is supported by the IR spectroscopy data. In the IR spectra of **Z1** + Cu and **Z2** + Cu, compared with the spectra of the original **Z1**–**Z3**, a sharp decrease in the intensity, as well as high-frequency shifts of the stretching ν (C=N) and bending vibrations δ (NH, NH_2_) bands by 9–13 cm^−1^, can be observed. This indicates the interaction of the C=N and N–H groups of **Z1** and **Z2** with the Cu(II) ions. The spectrum of the initial **Z3** contains a band at 1651 cm^−1^, which is attributable to the C-O stretching vibration. The spectra of **Z3** + Ni and **Z3** + Co show low-frequency shifts in the C=O stretching vibration to 1640 cm^−1^ and 1642 cm^−1^, ∆ν = 9 cm^−1^ and 11 cm^−1^, correspondingly, after the adsorption of Ni(II) and Co(II).

The survey of the literature data and our own results demonstrate that the interaction between the metal ions (Cu, Co, Ni) and C=N, NH, NH_2,_ and C=O groups in silane–guanidines **1**–**3** is thermodynamically favorable. As a rule, this takes place through a chelate interaction and involves the generation of sustainable complexes [61,62].

### 2.6. NMR Spectroscopy

The modified zeolites **Z1**–**Z3** exhibited insolubility. Hence, a solution of the starting silanes **1**–**3** in CDCl_3_ was employed to record the NMR spectra. The monomers **1**–**3** shared similar structural components with **Z1**–**Z3**, thereby justifying their use as models.

The ^1^H and ^13^C NMR spectra of the initial **1**–**3** were studied. The ^1^H NMR spectra of **1**–**3** showed signals of OEt protons in the regions of 1.12–1.19 (CH_3_) and 3.77–3.78 (OCH_2_) ppm; multiplets of SiCH_2_, CH_2_, and NCH_2_ protons at 0.56–0.60, 1.58–1.60, and 3.19–3.25 ppm, correspondingly; and broadened signals of NH and NH_2_ protons at 5.90–8.50 ppm (only in DMSO). The signal at 1.92 ppm corresponded to the (CH_3_)C=O group in acetylguanidine–silane **3**.

The ^13^C NMR spectra of **1**–**3** showed OEt signals in the regions of ~18 and 60 ppm; SiCH_2_, CH_2_, NCH_2_, and carbon atoms were observed at ~5–7, 21–22, and 35–38 ppm, correspondingly; and the signal of the C=O carbon (in acetylguanidine–silane **3**) appeared at 172 ppm. The signals of C=N groups were not detected, probably due to broadening. The NMR spectra are shown in Figures S4–S6.

Thus, the NMR spectroscopy confirms the structure of guanidine–silanes **1**–**3**.

### 2.7. SEM-EDX Analysis

**Figure 3:**
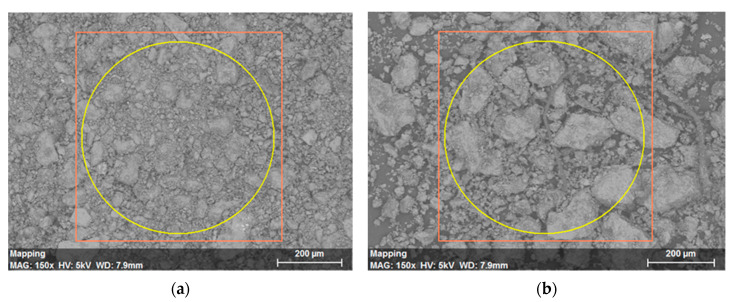
EM images: (**a**) **Z**; (**b**) **Z2**.

Scanning electron microscopy (SEM) is among the most powerful and broadly employed techniques for surface analysis. An SEM analysis was employed to visualize the coating on the zeolite **Z**. The SEM analysis confirmed the fixation of silanes **1**–**3** on the zeolite **Z** surface (see, for example, the SEM images of **Z** and **Z2** in Figure 3a,b).

As seen in Figure 3a,b, the chemical modification of the zeolite **Z** led to its structural changes. After modification, the surface of **Z2** became more uniform.

**Figure 4:**
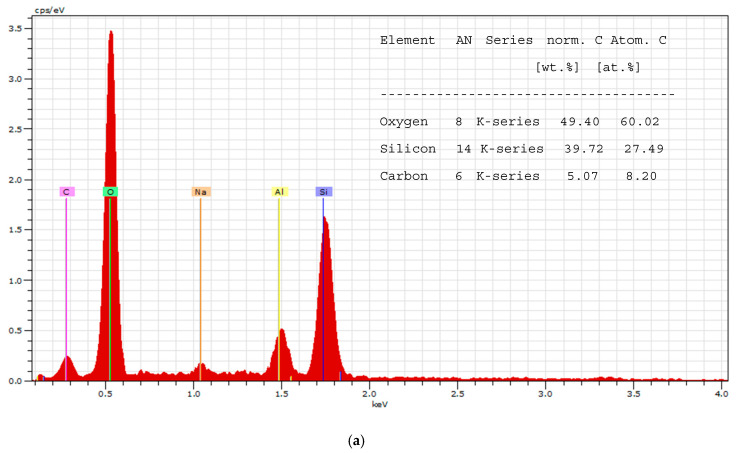

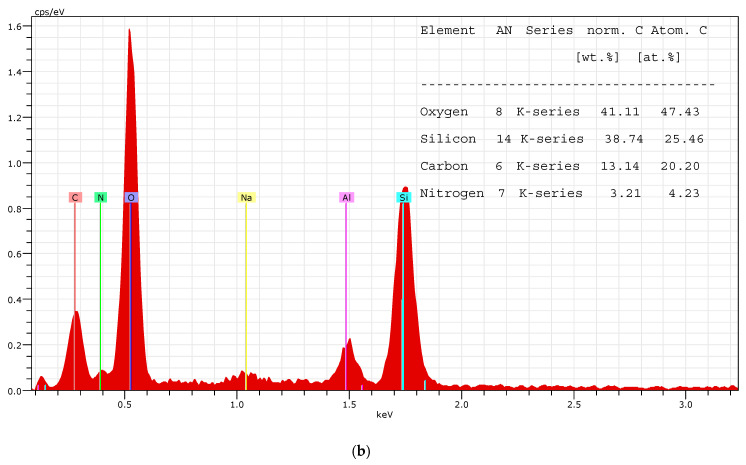
(**a**) EDX spectrum and elemental composition of zeolite **Z**; (**b**) EDX spectrum and elemental composition of modified zeolite **Z3**.

Energy-dispersive X-ray spectroscopy (EDX) is exploited to establish the elemental composition of a surface and gives an overall mapping of a sample. The large degree of **Z** coating, for example, with acetylguanidine–silane **3** was proved by the EDX analysis (compare Figure 4a,b).

It is known that heulandite **Z**, Ca[Al_2_Si_7_O_18_] 6H_2_O, does not contain nitrogen. The increased content of carbon (up to 13.14 wt.%) and nitrogen (up to 3.21 wt.%) on the surface of **Z3** indicates that the zeolite surface was coated with a silane **3** layer. A similar process took place when the zeolite **Z** was treated with silanes **1** and **2**. The high content of carbon (10.99–13.15 wt.%) and nitrogen (4.37–4.65 wt.%) on the surface of **Z1** and **Z2** shows that these zeolite surfaces were also covered with silane layers (Figures S7–S9). The SEM images, EDX spectra, and metal mapping for surfaces **Z1**–**Z3** are shown in Figures S10–S23.

The adsorption of Cu(II), Co(II), and Ni(II) ions did not induce noticeable structural alterations to the modified **Z1**–**Z3** surfaces (SEM image in Figure S10). At the same time, the EDX analysis confirms the presence of adsorbed Cu(II), Co(II), and Ni(II) ions on the surfaces of the modified zeolites **Z1**–**Z3** (Figures S11–S15). For example, after the adsorption of individual metals from the one-component solutions, the content of copper, cobalt, and nickel on **Z3** was 1.05 wt.%, 1.02 wt.%, and 0.75 wt.%, respectively.

Samples of zeolites **Z1**–**Z3** from the three-component solutions were studied after adsorption. The EDX analysis shows that these samples adsorbed all the ions, but in different manners. For example, sample **Z3** + Cu + Co + Ni contained Cu 0.52 wt.%, Co 0.31 wt.%, and Ni 0.06 wt.% (Figure S23). **Z1** + Cu + Co + Ni included Cu 1.21 wt.%, Co 0.54 wt.%, and Ni 0.02 wt.% (Figure S16). All this demonstrates that the modified zeolites can be appropriate adsorbents for each of the above metals, but to different extents. The degree of adsorption decreases in the order of Cu ˃ Co ˃ Ni. This fact is consistent with the adsorption isotherms and adsorption capacity values (Figure 2a).

In addition, the study of the element distribution maps on the surfaces of modified zeolites **Z1**–**Z3** before and after adsorption surprisingly revealed the superposition of maps. This confirms the co-localization of active ligand atoms (nitrogen atoms in guanidine fragments) and metal ions (Figure S24).

Earlier, it was shown that nitrogen-rich guanidines are becoming a more and more popular and valuable class of ligands and proligands. They are frequently found in natural and biologically (pharmacologically) active compounds and ensure a variety of ways to stabilize and coordinate transition metals [53,54,55,56,66,67,68,69,70].

**Scheme 8:**
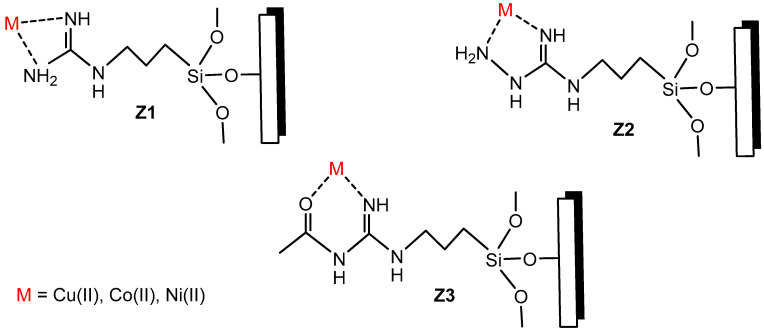
Generation of chelate complexes of metal ions on **Z1**, **Z2**, and **Z3** surfaces.

Based on our data (adsorption isotherms; FT-IR, NMR, and EDX spectra; and SEM images) and the literature findings [66,67,68,69,70], it can be assumed that the adsorption of heavy metal ions onto the modified zeolites’ **Z1**’**s**–**Z3**’**s** surfaces involved the generation of chelate complexes (1:1) owing to the donor–acceptor interaction of Cu, Co, and Ni ions with the nitrogen atoms (nitrogen and oxygen) of the guanidine (**Z1**), aminoguanidine (**Z2**), and acetylguanidine (**Z3**) fragments (Scheme 8).

Such coordination modes are typical for guanidine ligands. Nevertheless, the formation of 2:1 complexes cannot be completely ruled out [66,67,68,69,70].

As seen from Scheme 8, the guanidine zeolite **Z1** can form four-membered metal complexes. At the same time, zeolites **Z2**–**Z3**, containing additional donor atoms (N or O), produce more stable five and six-membered chelate complexes.

According to the EDX spectral data (Figures S11, S17 and S18) the content of Ni in zeolites **Z1**–**Z3** is 0.46 wt.%, 0.46 wt.%, and 0.75 wt.%, respectively. Of course, these results are only estimations, since EDX is a semi-quantitative analytical approach. However, a preliminary conclusion can be drawn: zeolite **Z3**, modified with acetylguanidine (additional functional group C=O), adsorbs metal ions 1.6 times better than **Z1** and **Z2**.

Thus, in the case of adsorption of Cu(II), Co(II), and Ni(II) ions by samples **Z1**–**Z3**, surface complexation occurs. Meanwhile, the negatively charged AlO_2_^−^ groups of the zeolite can promote adsorption. Finally, interactions between the metal ions and the hydroxo groups of zeolite, as well as exchanges with Ca (II) ions, cannot be completely excluded. However, our investigations demonstrate that the adsorption features of adsorbents **Z1**–**Z3** are similar and often exceed those of zeolites modified with functionalized organosilicon and organic materials.

## 3. Materials and Methods

### 3.1. Materials

The work focused on natural zeolite sourced from the Kholinsky deposit (LLC “Zeolite-Trade”, St. Petersburg, Russia), known as calcium heulandite (Ca[Al_2_Si_7_O_18_] 6H_2_O), and denoted as **Z**. This zeolite typically contains 25–30 wt.% rock impurity, specifically KAlSi_3_O_8_. Following the classification of D. Breck [71], heulandite, akin to clinoptilolite, falls within group 7 of plate-like zeolites, characterized as microporous sorbents with micropore dimensions ranging from 0.5 to 1.5 nm.

Commercially available materials, including CuSO_4_ 5H_2_O from Merck (Darmstadt, Germany), CoCl_2_ 6H_2_O from Merck, NiSO_4_∙7H_2_O from Merck, (NH_4_)_2_SO_4_ from Merck, 1-(3-aminopropyl)-triethoxysilane from Sigma-Aldrich, guanidine carbonate from Aldrich (St. Louis, MO, USA), 1-acetylguanidine from Aldrich, and hexane from Sigma (St. Louis, MO, USA), were utilized as received.

The reference solutions, containing ions of Cu(II), Co(II), and Ni(II) at 10 mg/mL concentrations of metals, were made by dissolving an appropriate amount of compound in deionized water. The solutions were subsequently diluted to obtain the desired concentrations for the adsorption experiments.

### 3.2. Methods

#### 3.2.1. Synthesis of N-[3-(Triethoxysilyl)propyl]guanidine (**1**)

Compound **1**, N-[3-(triethoxysilyl)propyl]guanidine, was prepared via condensation of guanidine carbonate with 1-(3-aminopropyl)triethoxysilane, as outlined in Scheme 3.

#### 3.2.2. Synthesis of N-[3-(Triethoxysilyl)propyl]aminoguanidine (**2**)

Compound **2**, N-[3-(triethoxysilyl)propyl]aminoguanidine, was prepared via condensation of aminoguanidine carbonate with 1-(3-aminopropyl)triethoxysilane, as depicted in Scheme 4.

#### 3.2.3. Synthesis of N-[3-(Triethoxysilyl)propyl]acetyl Guanidine (**3**)

Compound **3**, N-[3-(triethoxysilyl)propyl]acetyl guanidine, was synthesized in similar manner, as illustrated in Scheme 5.

#### 3.2.4. Immobilization of Silanes (**1**), (**2**), and (**3**) on Zeolite

The procedure for immobilizing silanes **1**, **2**, and **3** onto the zeolite surface was conducted as per the protocol described in the literature [52]. Initially, a mixture of zeolite **Z** (1 g, fraction of particle size 0.5–1.0 mm) and hexane (90 g) was prepared. Subsequently, 1 g of either **1**, **2**, or **3** was added to the mixture and stirred for 10 min. The mixture was then heated (50 °C) and stirred for an additional hour. Afterward, the resulting product was filtered and washed successively with hexane and EtOH to eliminate any excess **1**, **2**, or **3**. The zeolites with immobilized **1**–**3** were subsequently dried at 110 °C for 1 h. These resulting zeolites, designated as **Z1**, **Z2**, and **Z3**, underwent characterization and were utilized to adsorb the ions of Cu(II), Co(II), and Ni(II) from water solutions.

#### 3.2.5. Evaluation of the Textural Properties of **Z** and **Z1**–**Z3**

The specific surface area and porosity and were assessed using a Brunauer–Emmett-Teller (BET) analysis conducted by SORBTOMETR-M and TERMOSORB devices (Group of companies “GRANAT”, St. Petersburg, Russia). The latter are specifically designed for investigating the textural properties of compounds. The performance of these analytical devices involves the adsorption/desorption of the gas adsorbate (N_2_) from the surface of the material.

According to this approach, the continuous flow of a gas mixture (He/N_2_) with a predetermined formulation is fed via the adsorber containing the material. During the adsorption/desorption, the proportion of the gas adsorbate in the composition alters, a change that is identified by a detector of thermoconductivity.

A signal produced by the detecting device indicates the peak, which is then transformed into an electric signal, signaling an elevated amount of gas adsorbate as it undergoes thermodesorption from the sample surface. The magnitude of the area beneath the peak correlates directly with the amount of gas adsorbate liberated from the sample during desorption.

#### 3.2.6. TGA-DSC Experiments

The TGA-DSC experiments were conducted using a STA 449 F1 Jupiter^®^ synchronous thermoanalyzer (NETZSCH-Gerätebau GmbH, Selb, Germany). The analysis involved heating a 5 g sample from 40 to 1000 °C at a 10 °C/min rate. This process was performed under an inert Ar atmosphere with a 70 mL/min flow rate. Corundum crucibles were used in the DSC analysis. During the experiment, a quadrupole mass spectrometer (QMS 403 C Aeolos, NETZSCH-Gerätebau GmbH, Selb, Germany) was utilized to monitor the quantitative and qualitative composition of gaseous products resulting from the thermal decomposition. The instrument operated with an energy of electron impact of 70 eV.

#### 3.2.7. Determination of Adsorption

In the experiments, a zeolite Z sieved fraction of (0.5–1.0 mm) was utilized. Its ion adsorption was investigated in water solutions containing CuSO_4_ 5H_2_O, CoCl_2_ 6H_2_O, NiSO_4_ 7H_2_O, and deionized water. The heavy metal ions (HMIs) concentration ranged 5–100 mg/L. After the adsorbent was filtered, the equilibrium concentration of HMIs in each solution was determined using Flame Atomic Absorption Spectroscopy (FAAS) with a Varian Spectra Plus instrument (Varian Optical Spectroscopy Instruments, Mulgrave, VIC, Australia).

The zeolites’ adsorption characteristics with respect to the Cu(II), Co(II), and Ni(II) ions were investigated in static conditions. Isotherms of the adsorption were constructed by a method of constant weighed amounts (0.1 g of zeolite), as well as variable concentrations of metal ions. The value of adsorption (A, mg/g) was obtained using Equation (2):

A = (C_0_ − C_eq_) V/m (2)

where C_0_ and C_eq_ are the starting and equilibrium concentrations of metal ions in a solution, mg/L; m is the adsorbent mass, g; and V is the solution volume, L.

The volume of solution equaled 0.05 L. To investigate the isotherms, the runs concerning the elimination of the ions of Cu(II), Co(II), and Ni(II) were repeated, and the mean value was employed for the calculations.

The zeolite adsorption characteristics were assessed in solutions containing multiple ions of metals, specifically Cu(II), Co(II), and Ni(II). The solutions were standardized using the same amount of each metal (100 mg/L), utilizing 0.1 g adsorbent mass and 100 mL solution volume. The time of contact time was fixed at 1 h, with the pH adjusted to 5, and the temperature held constant at 298 K.

The pH values of the model solutions were measured on a pH-340 potentiometer (Measuring Instruments Plant, Gomel, Belarus) in accordance with the typical procedures. Thermal control was achieved with a UTU-4 thermostat, maintaining a constant temperature of 298 K throughout the studies. The stirring of the solutions was conducted at 1500 rpm with a magnetic stirrer, and stirring conditions were maintained across all the experiments.

The equilibrium data from the adsorption were analyzed by applying the isotherm model of Dubinin–Radushkevich:

*A* = *A*_m_
*exp*(−*k* ɛ^2^) (3)

where *k* is a constant (mol^2^/kJ) associated with the adsorption energy and ε represents the Polanyi potential (kJ/mol), which characterizes the isothermal work required to transfer 1 mol of HMIs from the bulk of the equilibrium solution to the adsorbent surface. This potential is determined using the following equation:

ε = *RT* ln (1 + 1/*C*) (4)

where R is the universal gas constant, J/mol K; and T is the absolute temperature, K.

The Dubinin–Radushkevich Equation (3), expressed logarithmically, is given by the following:

ln *A* = ln *A*_m_ *− k* ε^2^ (5)

The experimental data were evaluated using the linear Langmuir’s equation:

1/*A* = 1/*A*_∞_ + ((1/(*A*_∞_·*K*) (1*/C_eq_*) (6)

where *A* is the value of current adsorption, mmol/g; and *A*∞ is the limiting adsorption value, mmol/g;

*K* is the constant of adsorption equilibrium; and *C_eq_* is the metal ion equilibrium concentration, mmol/L.

The Gibbs free energy was measured using the Van’t Hoff isotherm equation, which is given by

Δ*G*^0^ = −*RT* ln *K* (7)

Furthermore, the empirical Freundlich equation is commonly employed to describe the adsorption process on a heterogeneous surface:

*A* = *K*_F_ + *C*_eq_ ^1/*n*^ (8)

where *A* is the adsorption value, mmol/g; *C*_eq_ is the equilibrium concentration of a heavy metal ion, mmol/L; and *K*_F_ and *n* are constants.

The experimental results were analyzed using the logarithmic type of the Freundlich equation:

log *A* = log *K*_F_ + 1/n log *C*_eq_ (9)

#### 3.2.8. H, ^13^C NMR, and FT-IR Spectroscopy and SEM-EDX Analysis

The ^1^H and ^13^C NMR spectra were run on Bruker DPX-400 spectrometers (Bruker, Karlsruhe, Germany), with operating frequencies of 400.13 MHz and 100.61 MHz, correspondingly. The measurements were conducted in CDCl_3_ at room temperature. The chemical shifts were calibrated with respect to TMS for both the ^1^H and ^13^C spectra.

The FT-IR spectra of zeolites were acquired using a Specord IR-75 spectrometer (Analytik Jena, Jena, Germany), specifically the Varian 3100 FT-IR model. The sorbents’ morphology was analyzed using scanning electron microscopy (SEM) with a Hitachi TM3000 electron microscope (Hitachi High-Tech Corporation, Tokyo, Japan). The instrument allows for magnification of up to 30,000× and offers a resolution of up to 25 nm. Operating at 5 kV, this setup enabled the observation of defects, such as thin films on the surface under investigation. Moreover, bulky compounds having shadow and volumetric contrast were identified by the backscattered electron detector. The elements on the surface were determined through an evaluation of energy-dispersive X-ray (EDX) spectra using a Quantax 70 system. The samples were scanned using a Quanta 200 FEI SEM-EDX electron microscope (FEI Company, Eindhoven, The Netherlands).

## 4. Conclusions

N-[3-(triethoxysilyl)propyl]guanidine (**1**), -aminoguanidine (**2**), and -acetylguanidine (**3**) were synthesized by the condensation of 1-(3-aminopropyl)triethoxysilane with the corresponding guanidines. The composition and structure of silanes **1**–**3** were confirmed by FT-IR and ^1^H and ^13^C NMR spectroscopy methods.

Silanes **1**–**3** were used to modify (silanize) the surface of natural “heulandite,” a zeolite (**Z**). Modified (functionalized) materials (**Z1**), (**Z2**), and (**Z3**) were obtained.

The properties and characteristics of the modified zeolites **Z1**–**Z3** were studied by adsorption/desorption (BET), SEM-EDX, and TGA-DSC methods. The zeolites **Z1**–**Z3** were used to evaluate the absorption of the following heavy metal ions: Cu(II), Co(II), and Ni(II). It was experimentally established that the adsorption activity of the modified zeolites (**Z1**–**Z3**) was 3–6 times larger than that of the starting zeolite (**Z**).

The adsorption models of Dubinin–Radushkevich, Langmuir, and Freundlich were employed to theoretically describe the process under study. The latter model turned out to be the most suitable. Both the experimental and theoretical examinations confirmed that the degree of adsorption decreased in the order Cu ˃ Co ˃ Ni.

The EDX spectra and elemental mapping indicated the formation of monolayers, such as **Z1** + Ni, **Z2** + Co and **Z3** + Cu, as well as mixed layers, **Z1** + Cu + Co + Ni and **Z3** + Cu + Co + Ni. This confirmed that the modified zeolites **Z1**–**Z3** were effective adsorbents for the extraction of metals, both in single- and multi-component systems.

Based on the data obtained, it can be assumed that adsorption occurred due to the generation of chelate complexes between the metal ions and functional moieties of guanidine (**Z1**), aminoguanidine (**Z2**), or acetylguanidine (**Z3**) fragments.

Thus, based on natural zeolite (**Z**) and silanes (**1**–**3**), modifications (**Z1**–**Z3**) were obtained for the first time. These functional materials are promising adsorbents of noble, heavy, and toxic metal ions and could find applications in industry, ecology, agriculture, medicine, and biology, and chemistry. They could be employed in various processes, such as water and soil remediation, chemosorption, filtration, and chromatography.

## Figures and Tables

**Table 1:** Degree of **Z** modification with silanes **1**–**3** (initial weight of **Z**, 10 g; solvent: hexane).

Mass of Silanes 1–3 in Solution, %	Mass of Z1; Z2; Z3, g	Mass Gain, g	Mass of 1–3 in Z1; Z2; Z3, %
0.5	10.33; 10.36; 10.39	0.33; 0.36; 0.39	3.2; 3.5; 3.7
1.0	10.54; 10.55; 10.59	0.54; 0.55; 0.59	5.1; 5.2; 5.5
2.0	10.95; 10.97; 10.99	0.95; 0.97; 0.99	8.7; 8.8; 9.1
3.5	11.84; 11.86; 11.87	1.84; 1.86; 1.87	15.5; 15.6; 15.8
4.5	11.98; 12.00; 12.00	1.98; 2.00; 2.02	16.6; 16.6; 16.8
5.5	12.10; 12.12; 12.13	2.10; 2.12; 2.14	17.3; 17.5; 17.7

**Table 2:** Surface characteristics of **Z** and **Z1**–**Z3**.

Characteristics	Z	Z1	Z2	Z3
Specific surface area, m^2^/g	32.8	5.7	5.4	5.8
Specific pore volume, cm^3^/g	0.015	0.003	0.005	0.006
Micropore volume, cm^3^/g	0.004	0.002	0.001	0.002
Average pore volume, nm	1.780	1.640	1.680	1.630
Apparent density, g/cm^3^	0.92	0.87	0.85	0.84

**Table 3:** Parameters of Dubinin–Radushkevich, Langmuir, and Freundlich models for adsorption of heavy metal ions (HMIs) by **Z3**.

**Dubinin–Radushkevich Model**					
HMI	Linear Form of the Dubinin–Radushkevich Equation	*A_m_*, mmol/g	*k*, mol^2^/kJ^2^	*E*, kJ/mol	*R* ^2^
Cu(II)	ln *A* = 0.416 − 0.0023·*ε*^2^	0.660	0.0023	14.9	0.998
Co(II)	ln *A* = 0.554 − 0.0029·*ε*^2^	0.577	0.0029	13.1	0.997
Ni(II)	ln *A* = 0.936 − 0.0033·*ε*^2^	0.392	0.0033	12.3	0.997
**Langmuir Model**					
HMI	Linear Form of the Langmuir Equation	*A_∞_*, mmol/g	*K*	Δ*G*^0^, kJ/mol	*R* ^2^
Cu(II)	1/*A* = 1.385 + 0.205 1/*C*_eq_	0.722	6.774	−21.82	0.995
Co(II)	1/*A* = 1.448 + 0.336 1/*C*_eq_	0.691	4.312	−20.70	0.994
Ni(II)	1/*A* = 1.923 + 0.644 1/*C*_eq_	0.520	2.987	−20.00	0.999
**Freundlich Model**					
HMI	Linear Form of the Freundlich Equation	*K*_F_		*n*	*R* ^2^
Cu(II)	log *A* = −0.093 + 0.512 log *C*_eq_	0.807		1.954	0.982
Co(II)	log *A* = −0.389 + 0.580 log *C*_eq_	0.408		1.724	0.975
Ni(II)	log *A* = −0.797 + 0.675 log *C*_eq_	0.160		1.481	0.987

## Data Availability

The dataset is available on request from the authors.

## Supplementary Materials

The following supporting information can be downloaded at: https://www.mdpi.com/article/10.3390/ijms26167903/s1.
