# Supplementary material for: Modification of the Zeolite Heulandite with N-(3-Triethoxysilylpropyl)guanidines Offers an Effective Approach to Enhancing Its Adsorption Capacity for Heavy Metal Ions
Source: Int J Mol Sci. 2025 Aug 15;26(16):7903. doi: 10.3390/ijms26167903 (PMC12386438; doi:10.3390/ijms26167903)
Supplement: Supplementary file 1 [file ijms-26-07903-s001.zip › ijms-3749452-supplementary.pdf]

Supplementary information

Modification of zeolite heulandite with N-(3-triethoxysilylpropyl)guanidines offers an effective approach to enhancing its adsorption capacity for heavy metal ions.

Sergey N. Adamovich [a,*], Arailym M. Nalibayeva [b], Yerlan N. Abdikalykov [b,c], Mirgul Zh. Turmukhanova [c], Elena G. Filatova [d], Alexandr D. Chugunov [d], Igor A. Ushakov [a], Elizaveta N. Oborina [a], Igor B. Rozentsveig [a,*], Francis Verpoort [e,f]

[a] *A. E. Favorsky Irkutsk Institute of Chemistry, SB RAS, 664033, Irkutsk, 1 Favorsky Str., Russia*
[b] *D.V. Sokolsky Institute of Fuel, Catalysis and Electrochemistry, 050010, Almaty, 142 D. Kunaeva Str., Kazakhstan*
[c] *Faculty of Chemistry and Chemical Technology, al-Farabi Kazakh National University 050040, Almaty, 71 Al-Farabi Avenue, Kazakhstan*
[d] *Irkutsk National Research Technical University, 664074, Irkutsk, 83 Lermontov Str., Russia*
[e] *State Key Laboratory of Advanced Technology for Materials Synthesis and Processing, Wuhan University of Technology, 430070, Wuhan, 122 Luoshi Road, China*
[f] *Joint Institute of Chemical Research (FFMiEN), Peoples Friendship University of Russia (RUDN University), 6 Miklukho-Maklaya Str., 117198 Moscow, Russia*

* Corresponding authors.
E-mail address: mir@irioch.irk.ru (S.N. Adamovich)
E-mail address: i_roz@irioch.irk.ru (I.B. Rozentsveig)

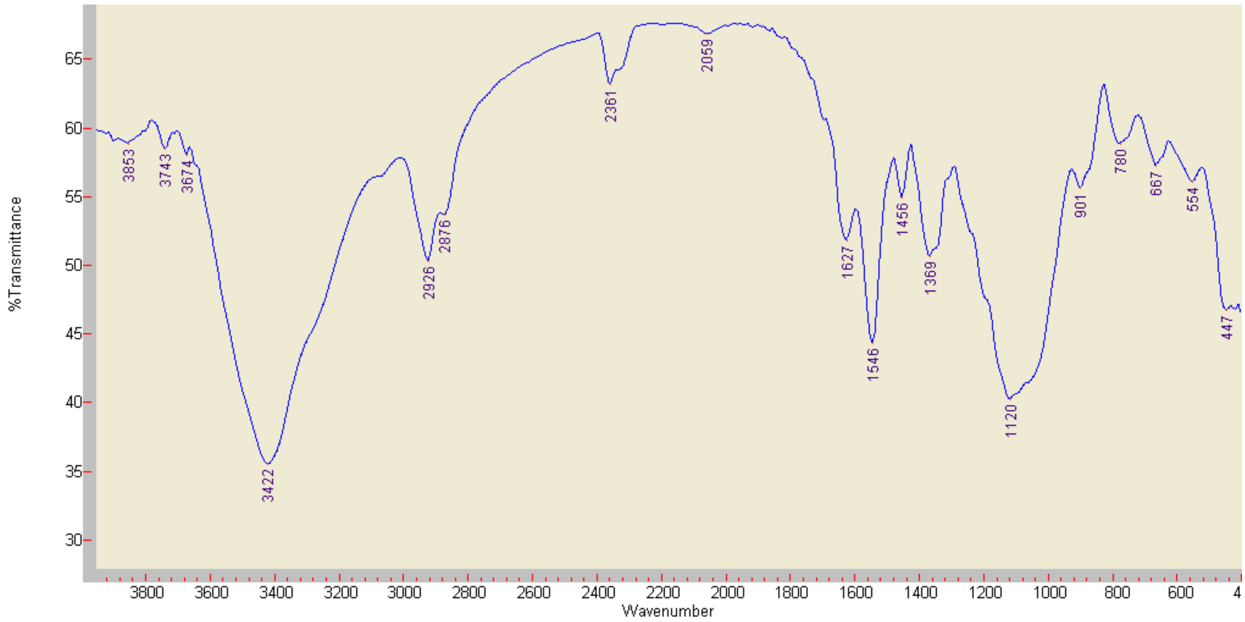

Figure S1. IR spectrum of modified zeolites **Z1**

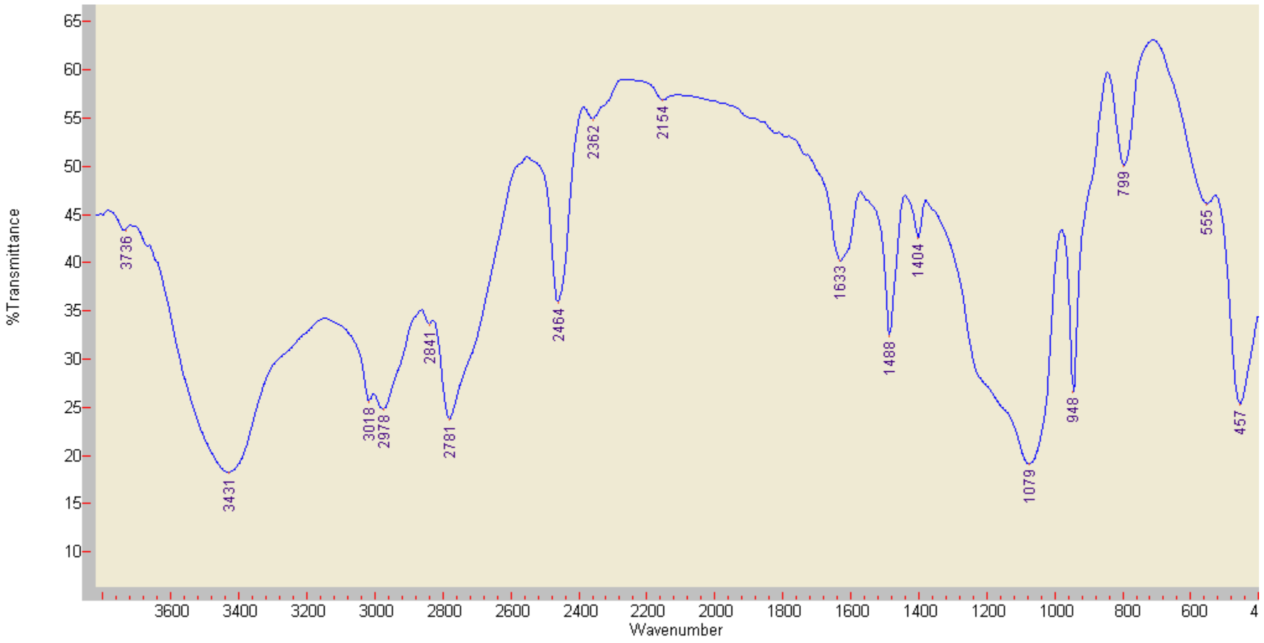

Figure S2. IR spectrum of modified zeolites **Z2**

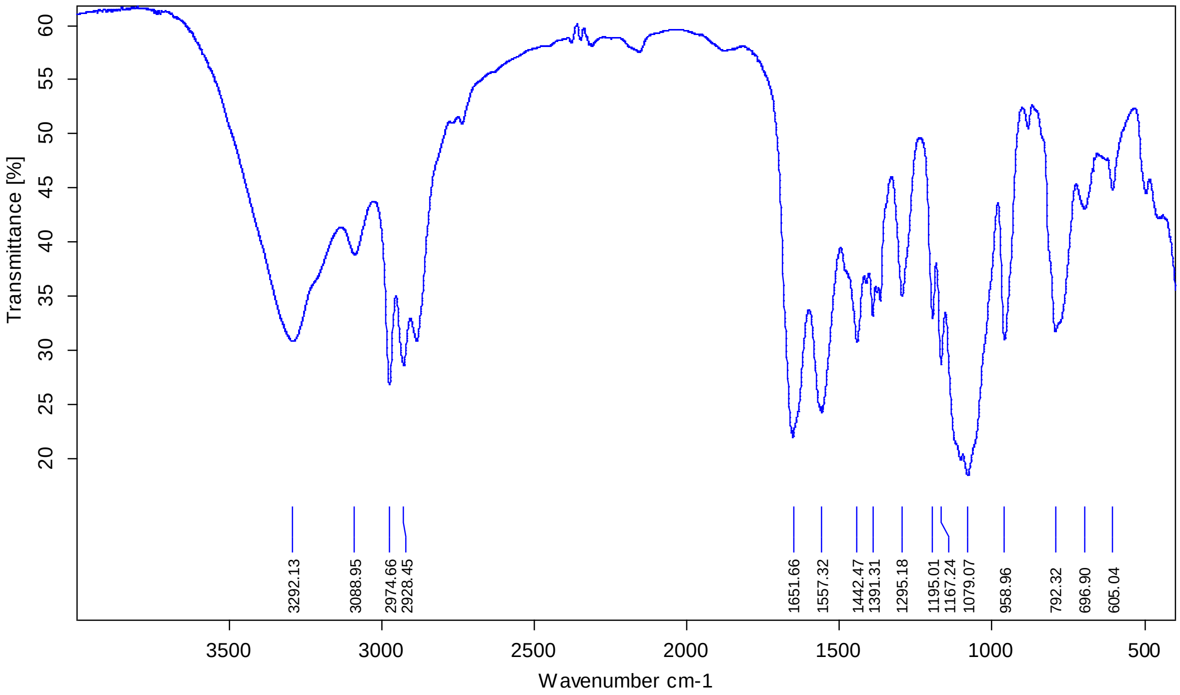

Figure S3. IR spectrum of modified zeolites **Z3**

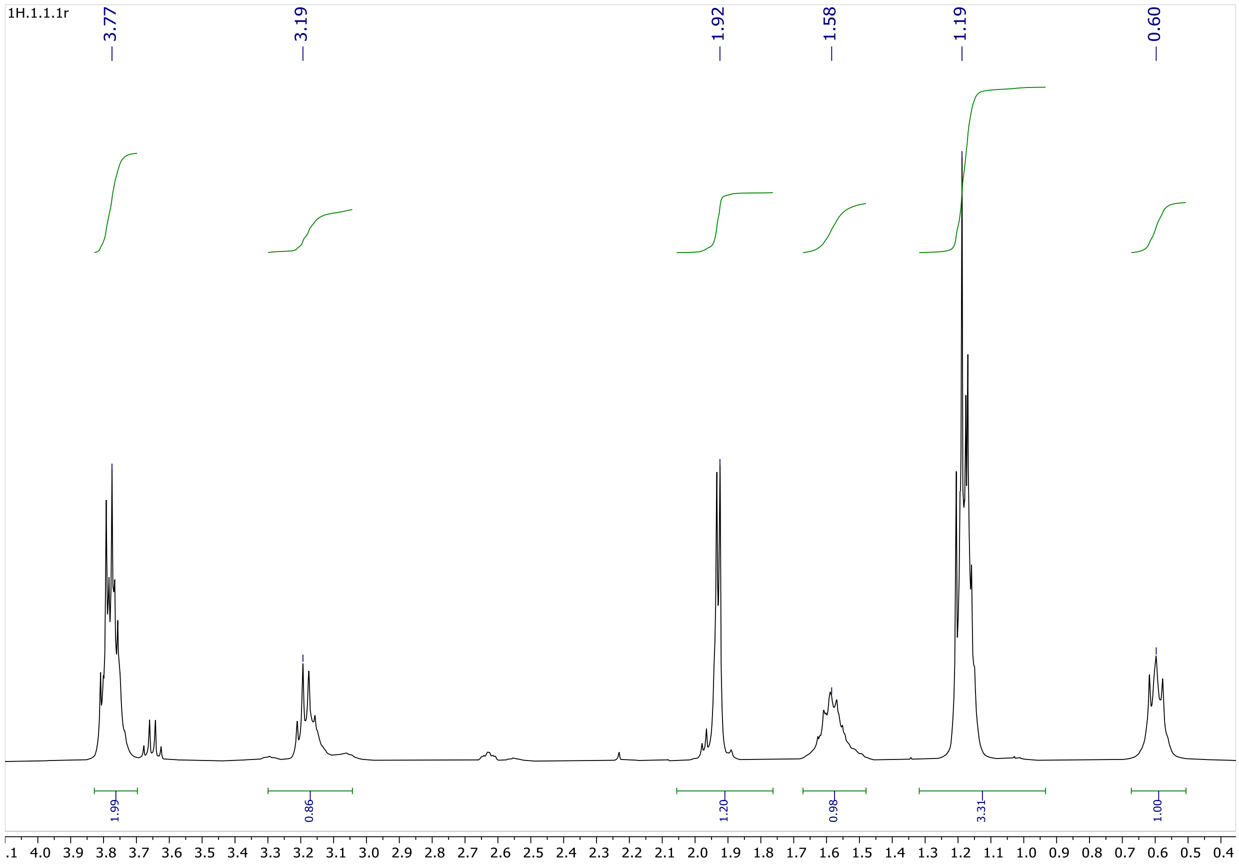

Figure S4. ^{1}H NMR spectrum of silane **3** ($CDCl_3$)

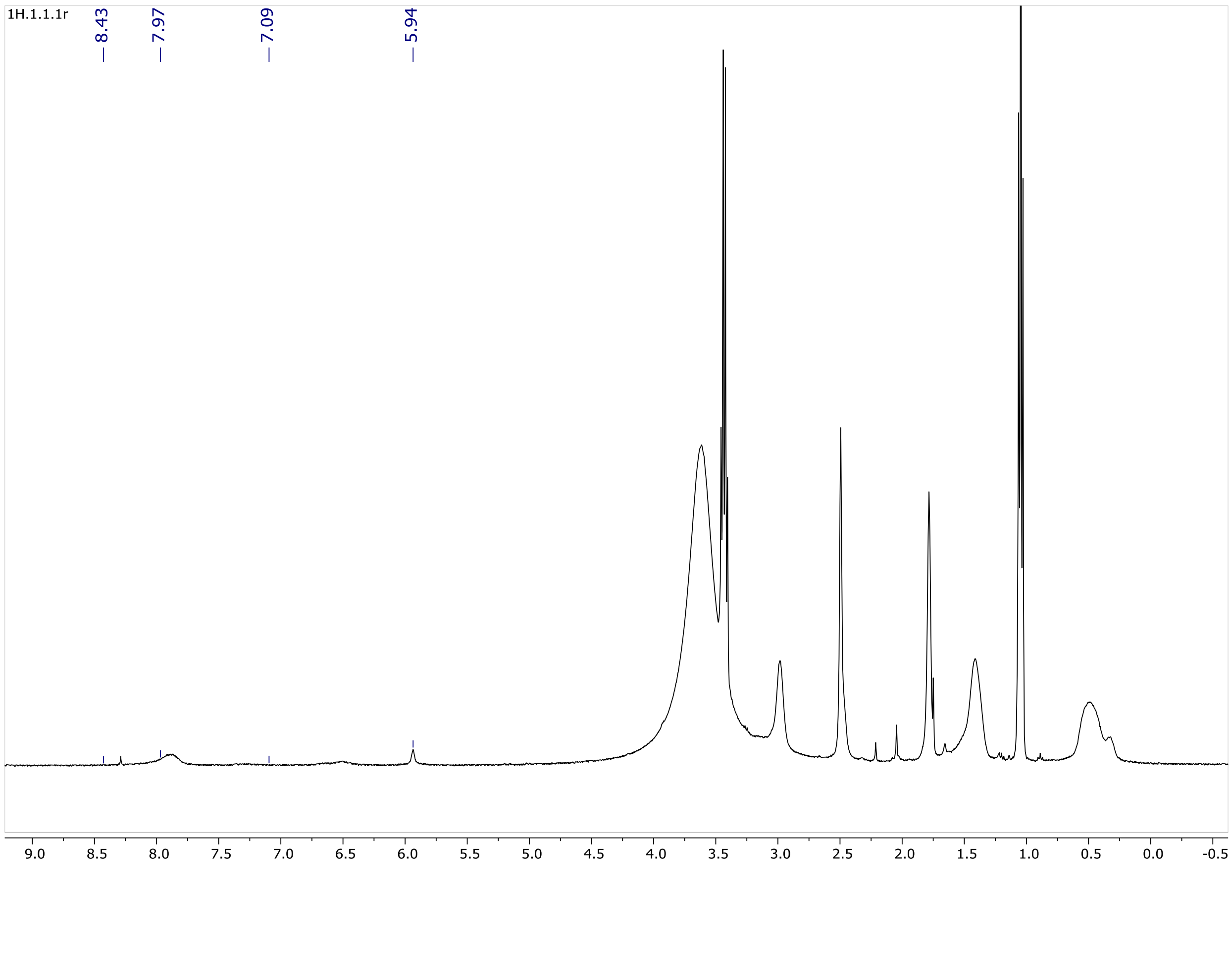

Figure S5. ^{1}H NMR spectrum of silane **3** (DMSO)

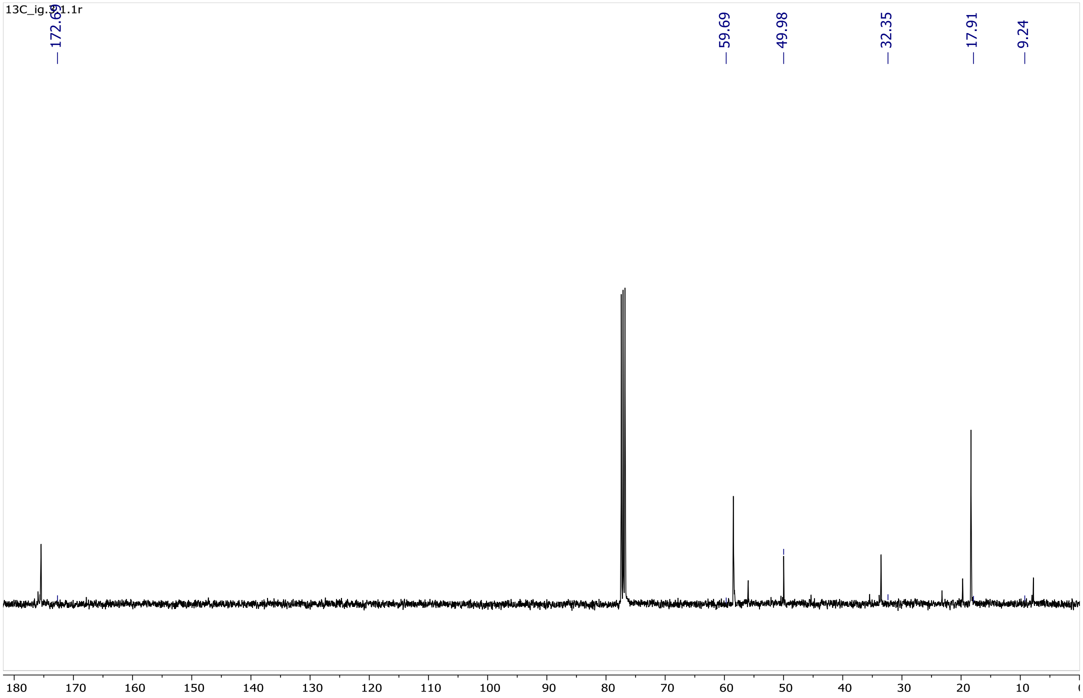

Figure S6. ^{13}C NMR spectrum of silane **3**

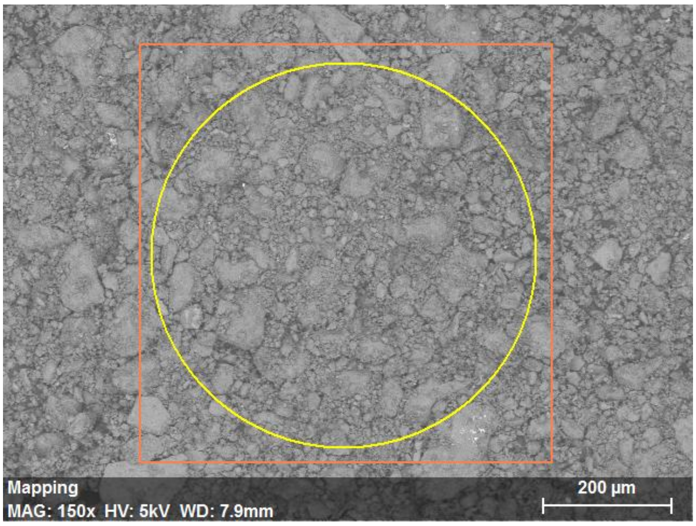

Figure S7a. SEM images of natural zeolite **Z.**

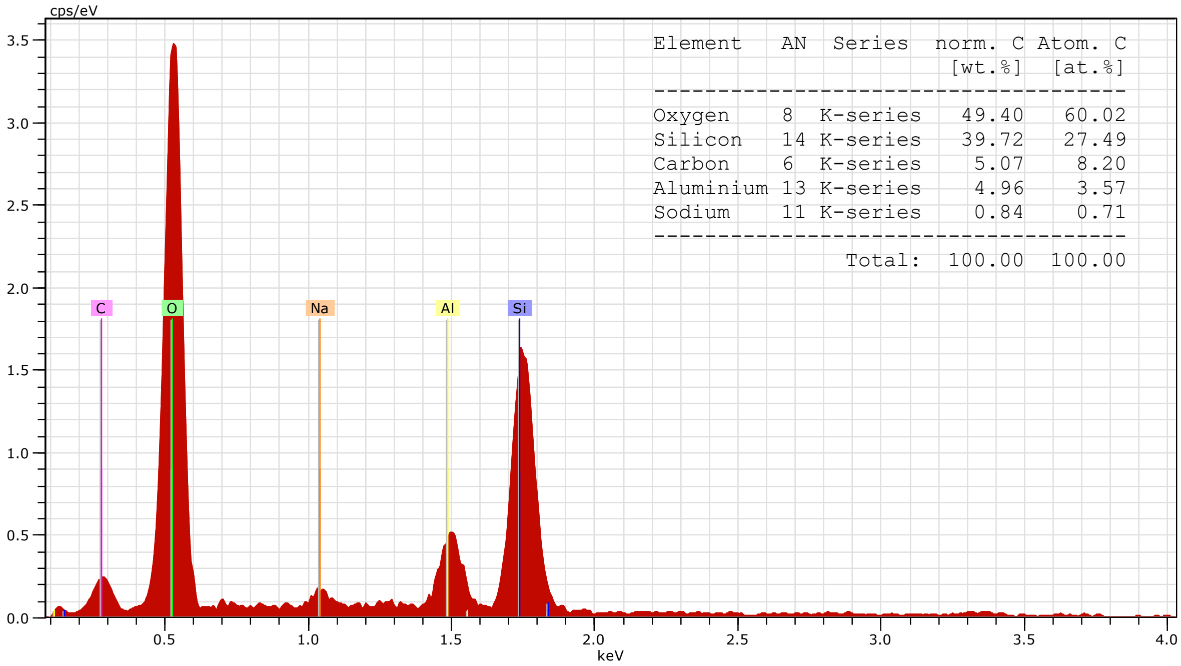

Figure S7b. EDX spectrum and elemental composition of natural zeolite **Z.**

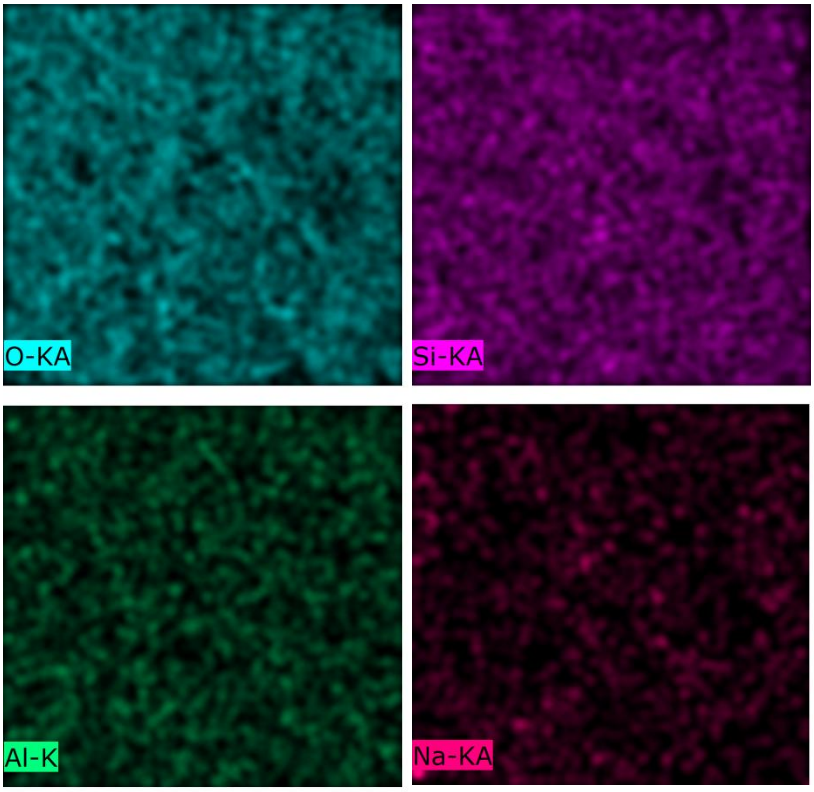

Figure S7c. Elemental mapping of natural zeolite **Z.**

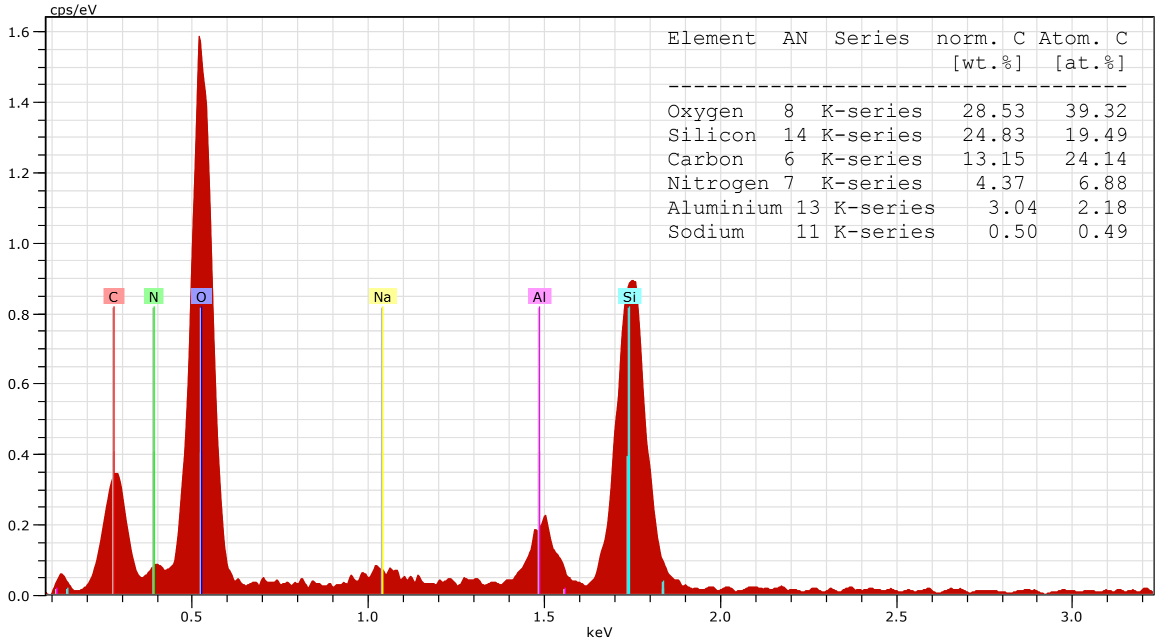

Figure S8a. EDX spectrum and elemental composition of **Z1**

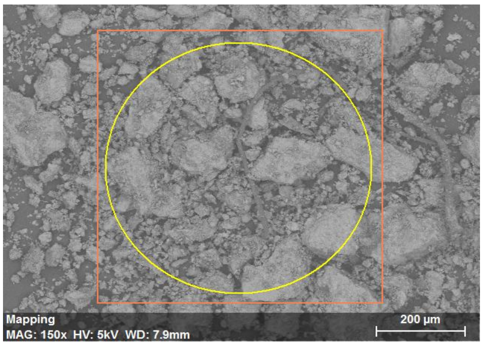

Figure S8b. SEM images of **Z2.**

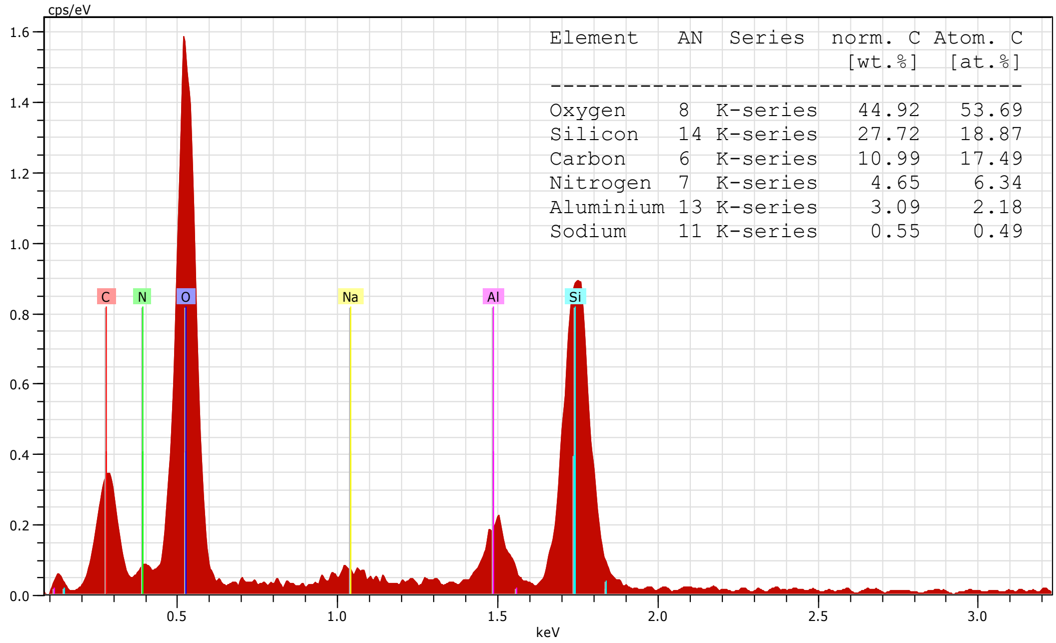

Figure S9. EDX spectrum and elemental composition of **Z2**

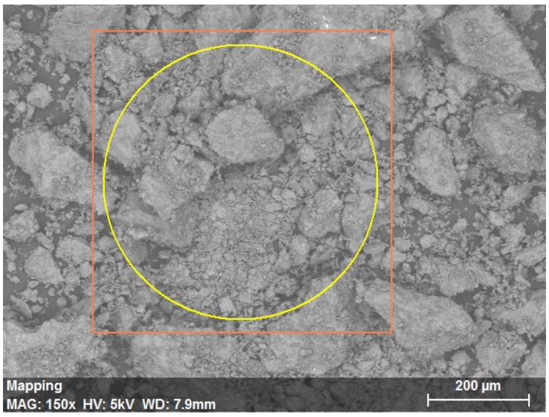

Figure S10. SEM images of **Z1+Ni**

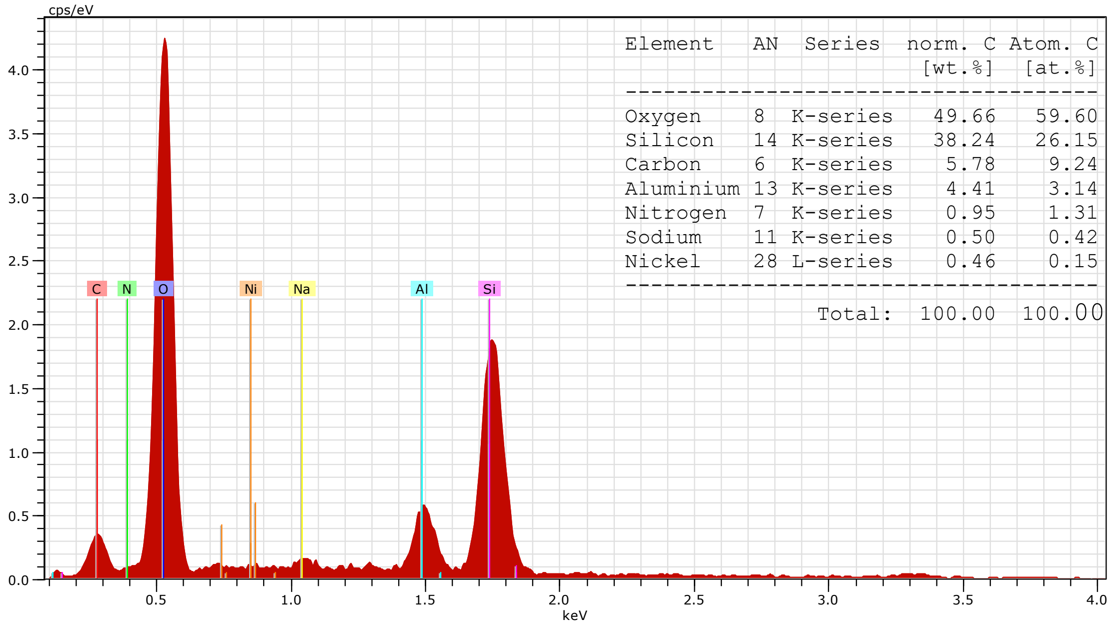

Figure S11. EDX spectrum and elemental composition of **Z1+Ni**

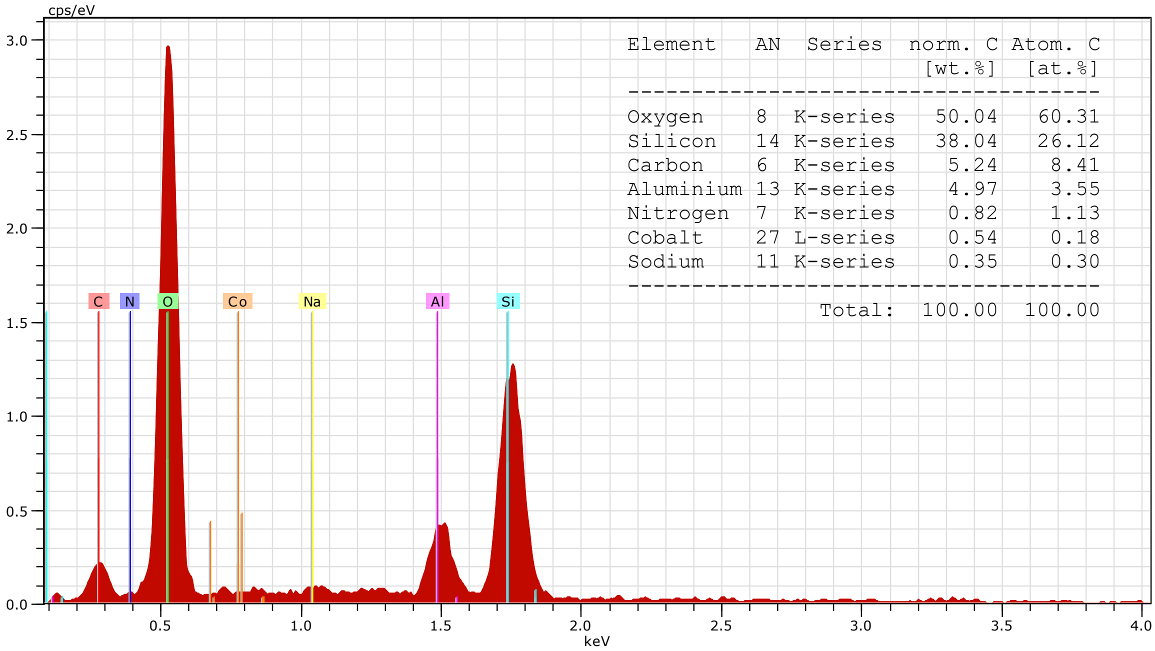

Figure S12. EDX spectrum and elemental composition of **Z1+Co**

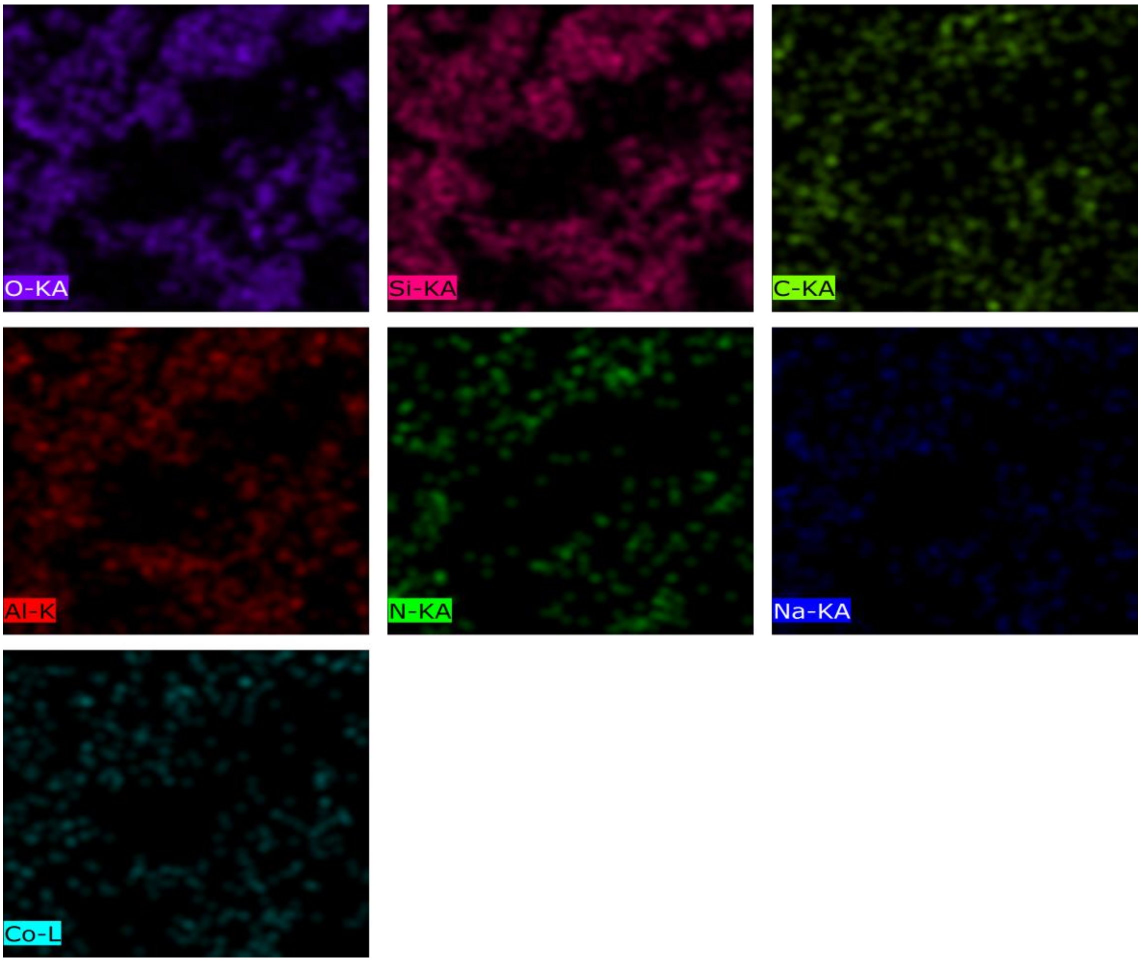

Figure S13. Elemental mapping of **Z1+Co**

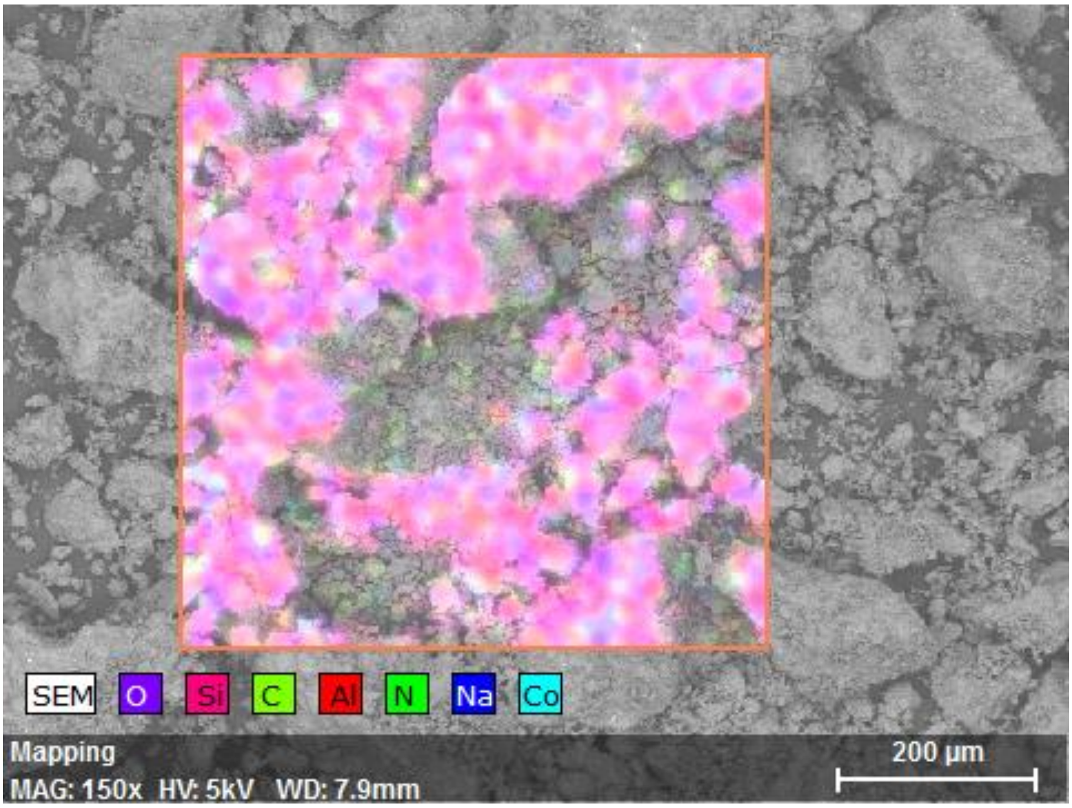

Figure S14. Elemental mapping of **Z1+Co**

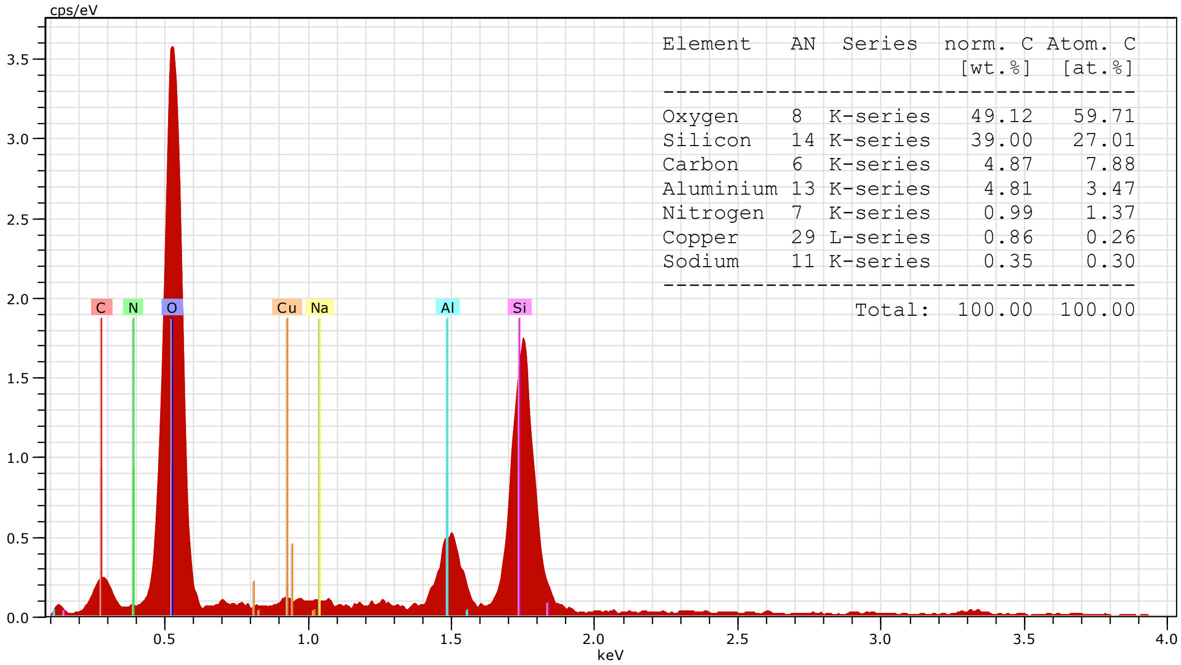

Figure S15. EDX spectrum and elemental composition of **Z1+Cu**

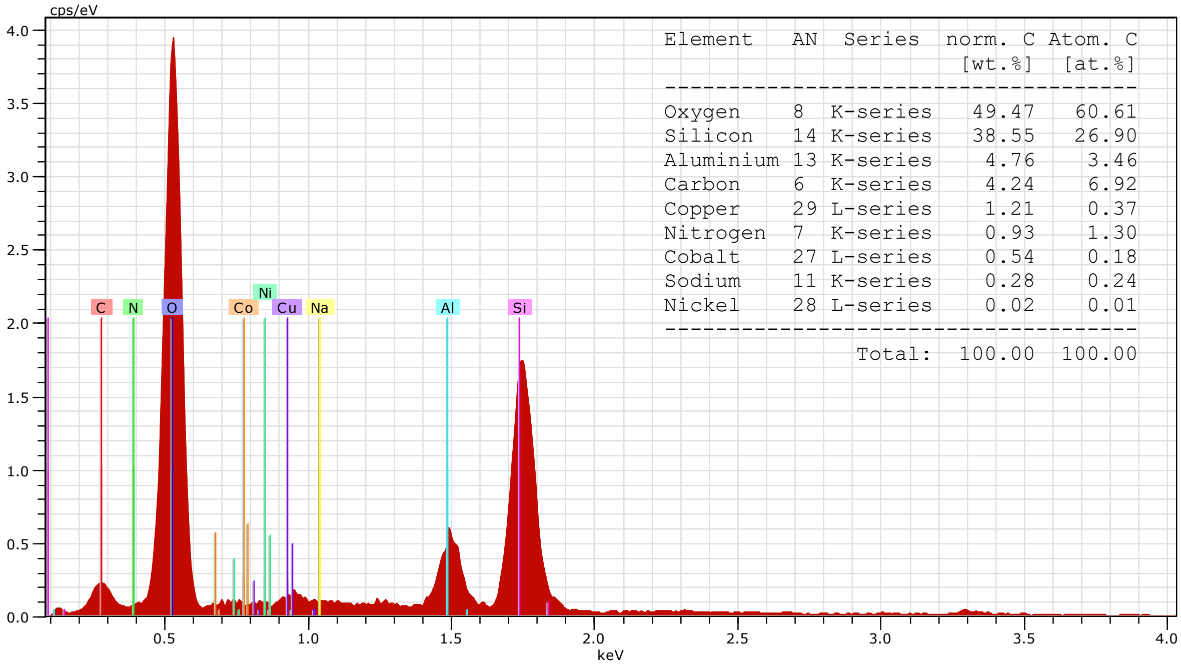

Figure S16. EDX spectrum and elemental composition of **Z1+Cu+Co+Ni**

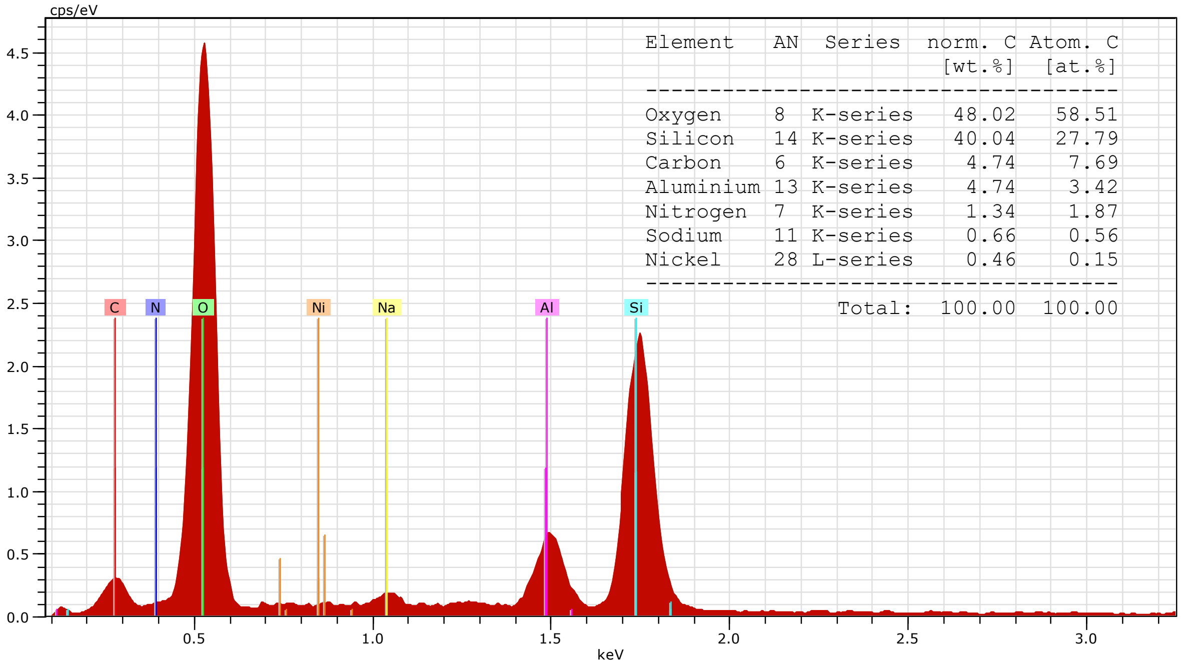

Figure S17. EDX spectrum and elemental composition of **Z2+Ni**

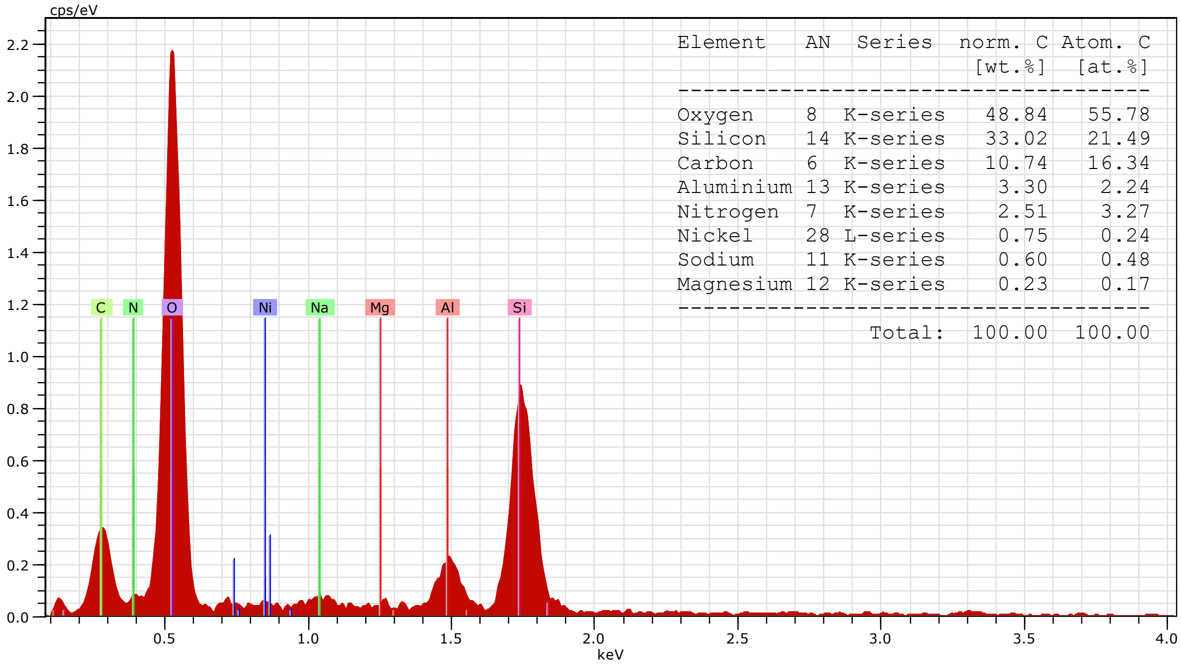

Figure S18. EDX spectrum and elemental composition of **Z3+Ni**

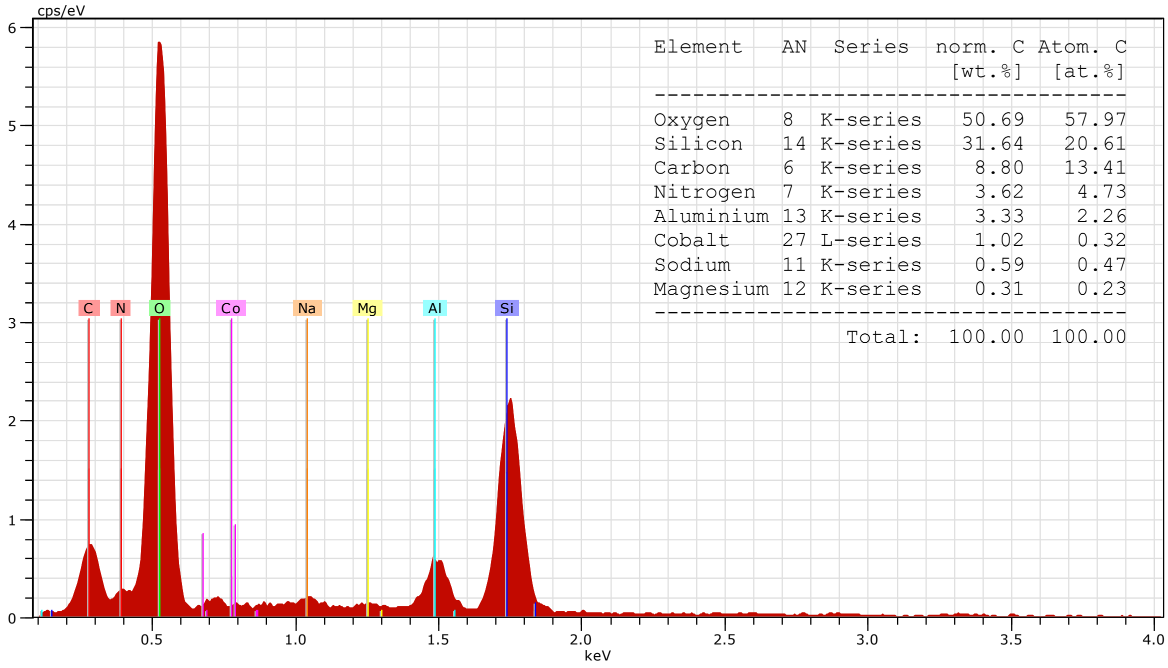

Figure S19. EDX spectrum and elemental composition of **Z3+Co**

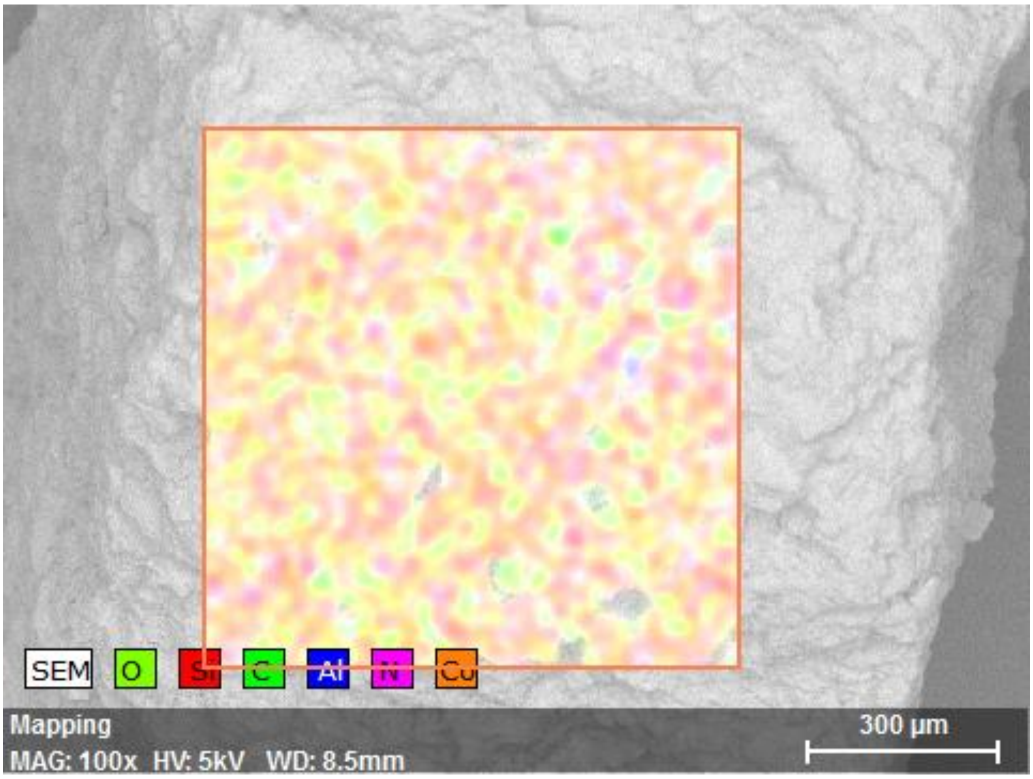

Figure S20. Elemental mapping of **Z3+Co**

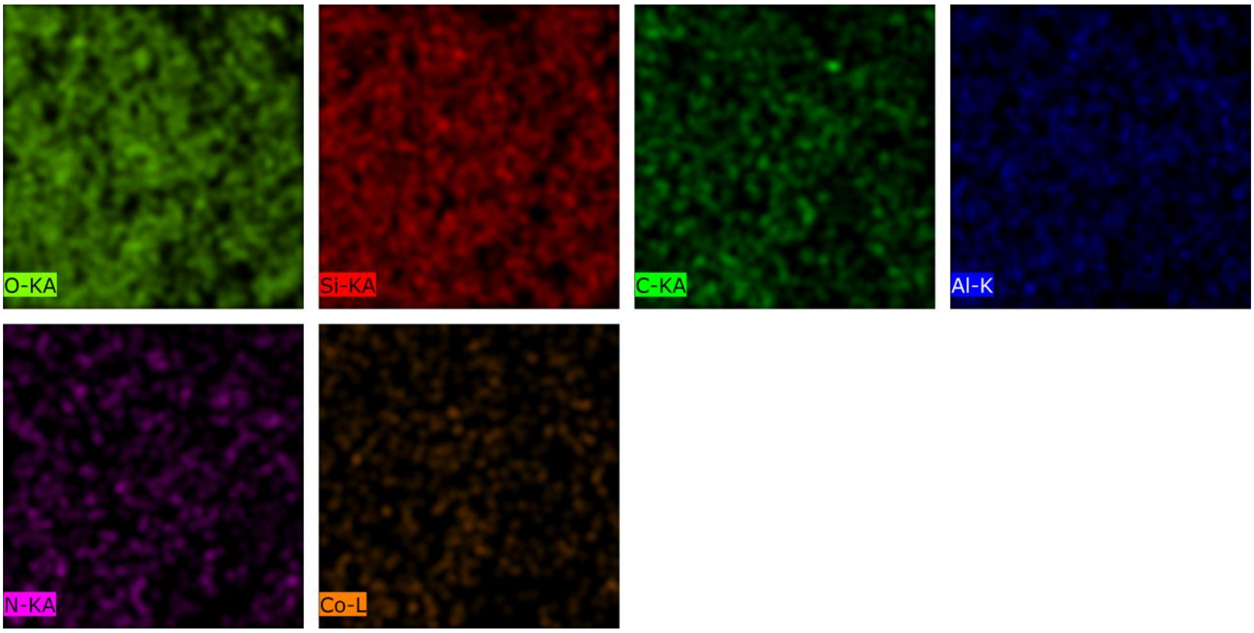

Figure S21. Elemental mapping of **Z3+Co**

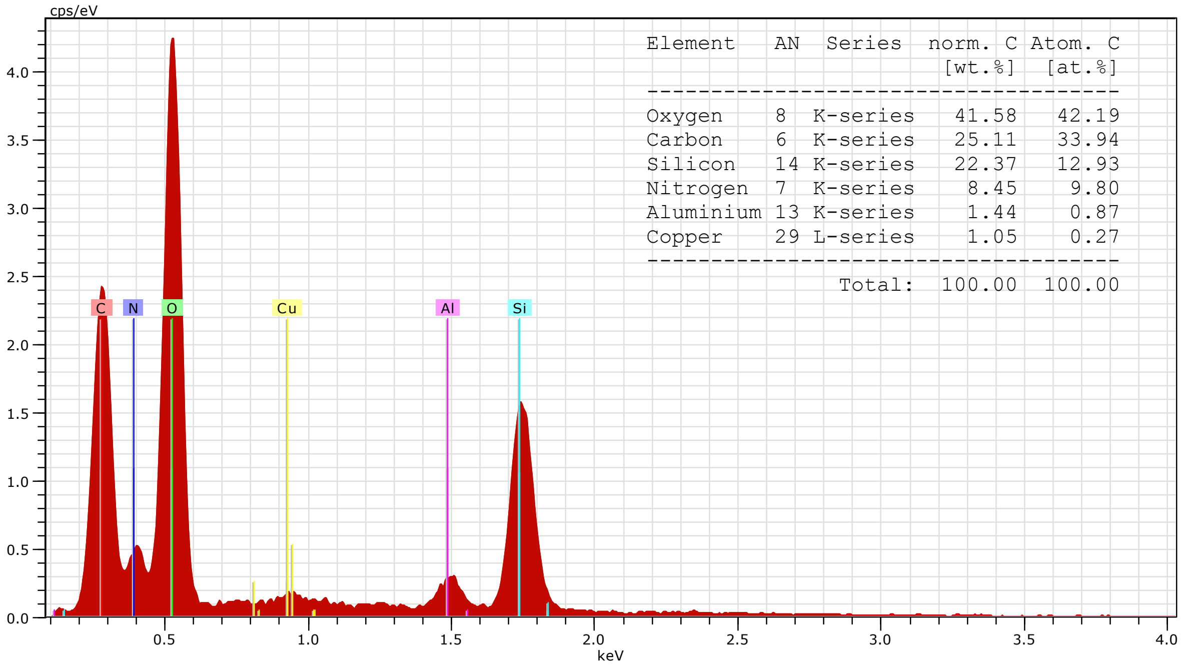

Figure S22. EDX spectrum and elemental composition of **Z3+Cu**

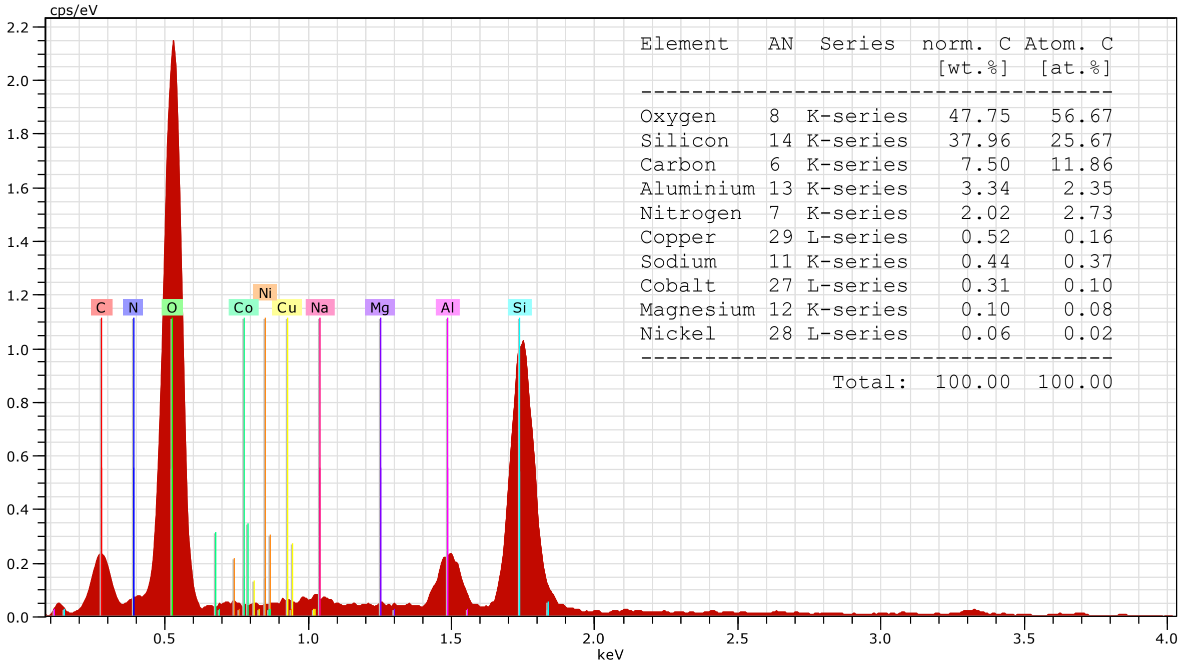

Figure S23. EDX spectrum and elemental composition of **Z3+Cu+Co+Ni**

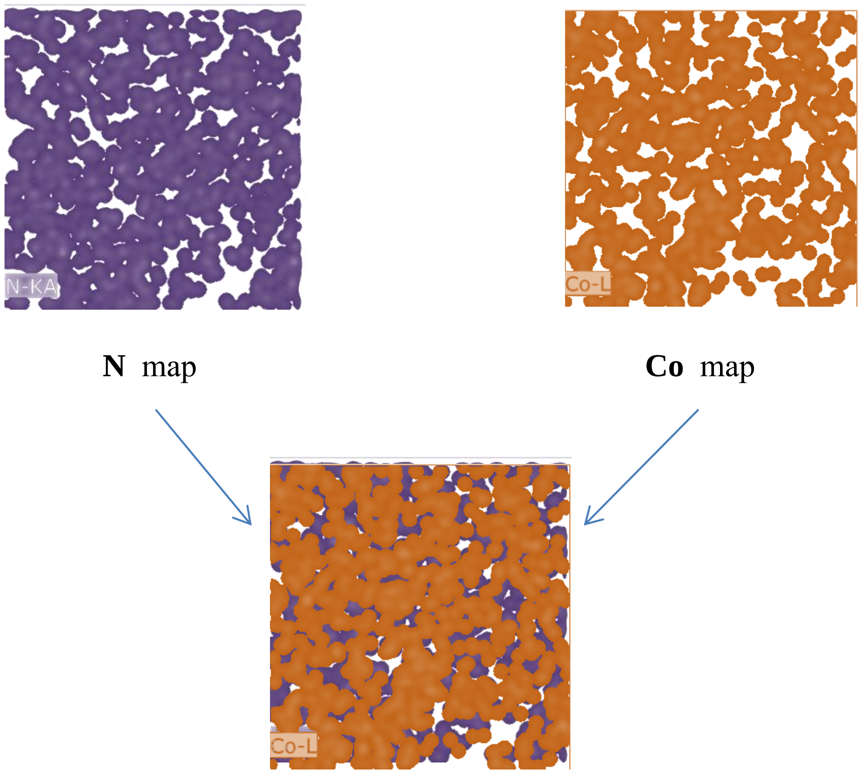

Superposition **N** and **Co** maps

Figure S24. Superposition **N** and **Co** maps of zeolite **Z3** after adsorption from **Co** solution
